# Salutary Effects of Overexpression of Rsm22, an Assembly Factor for the Mitochondrial Ribosome, on Frataxin/Yfh1 Depletion Phenotypes in *Saccharomyces cerevisiae*

**DOI:** 10.3390/biom15060785

**Published:** 2025-05-28

**Authors:** Ashutosh K. Pandey, Pratibha Singh, Jayashree Pain, Andrew Dancis, Debkumar Pain

**Affiliations:** Department of Pharmacology, Physiology and Neuroscience, New Jersey Medical School, Rutgers University, Newark, NJ 07103, USA; pandeyas@njms.rutgers.edu (A.K.P.); fp308@njms.rutgers.edu (P.S.); painja@njms.rutgers.edu (J.P.); andrewdancis@gmail.com (A.D.)

**Keywords:** yeast, mitochondria, frataxin, Yfh1, ferredoxin, iron–sulfur proteins, Rsm22, METTL17

## Abstract

Frataxin is a component of the iron–sulfur (Fe-S) cluster assembly complex in mitochondria, and deficiency is associated with Friedreich ataxia (FA). The yeast homolog Yfh1 resembles and cross-complements with its human equivalent, and frataxin bypass scenarios are of particular interest because they may point to strategies for treating FA. Here, we describe frataxin/Yfh1 bypass by overexpression of Rsm22, an assembly factor for the mitochondrial ribosome. Rsm22 overexpression in Yfh1-depleted yeast cells restored critical processes in mitochondria, including Fe-S cluster assembly, lipoic acid synthesis, iron homeostasis, and heme synthesis, to a significant extent. Formation of cytoplasmic Fe-S proteins was also restored, suggesting recovery of the mitochondrial ability to generate the (Fe-S)_int_ intermediate that is exported from mitochondria and is utilized for cytoplasmic Fe-S cluster assembly. Importantly, an essential component of the mitochondrial iron–sulfur cluster machinery, namely ferredoxin, was virtually absent in mitochondria lacking Yfh1, but it was recovered with Rsm22 overexpression. Interestingly, ferredoxin overexpression could offset some of the effects of Yfh1 depletion. Ferredoxin has recently been shown to bind to the cysteine desulfurase protein Nfs1 at the same site as Yfh1, in a conserved arginine patch on Nfs1, such that ferredoxin binding at this site may confer frataxin-bypass activity.

## 1. Introduction

Friedreich ataxia is an inherited neurodegenerative disease canonically affecting dorsal root ganglia, although other tissues are also impacted [1]. Importantly, cardiomyopathy can develop in patients, limiting longevity and causing much of the disease morbidity. The causative frataxin gene is highly conserved throughout evolution, and in the disease state, an expansion of GAA triplets in the first intron leads to impaired transcription and lowered frataxin protein levels [2,3]. The corresponding protein is localized primarily in the mitochondrial matrix and is associated with the mitochondrial inner membrane [4]. Mitochondrial frataxin, called Yfh1 in the yeast *Saccharomyces cerevisiae*, associates with a multi-subunit iron–sulfur (Fe-S) cluster assembly complex. The complex consists of two copies each of the cysteine desulfurase subcomplex Nfs1-Isd11-Acp1, the scaffold protein (Isu1/2), the electron donor ferredoxin (Yah1), and frataxin (Yfh1). The dysfunction of frataxin/Yfh1 causes Friedreich ataxia [5,6]. Of note, the ferredoxin Yah1 itself contains a [2Fe-2S] cluster, and under certain conditions, Yah1 may compete with frataxin/Yfh1 for overlapping binding sites on the Nfs1 core complex [7].

Fe-S clusters are inorganic cofactors consisting of iron and sulfur; they can form spontaneously in vitro, but that is not the way they are synthesized in cells. In cells, they are manufactured by a complex, highly regulated machinery [8]. In terms of function, they perform vital roles in numerous essential pathways, including cellular respiration, protein translation, iron sensing, enzymatic reactions, and DNA synthesis and repair [5]. After years of controversial research, a consensus has emerged regarding the function of frataxin, which is now thought to act as a facilitator of sulfur transfer in an early stage of Fe-S cluster assembly [9]. Nfs1 makes use of pyridoxal phosphate (PLP) chemistry to abstract sulfur from the amino acid cysteine, creating a sulfur adduct on a critical cysteine (Cys 421 in yeast Nfs1). A mobile swinging “arm” on Nfs1 carries the persulfide sulfur to a recipient cysteine on Isu (Cys 139 in yeast Isu1), transferring it in a regulated fashion. The frataxin molecule is located in the complex in proximity to the donor and recipient of the persulfide sulfur, and thus, it is able to act as a facilitator, optimally positioning assembly site residues for rapid sulfur transfer [10]. Iron for forming the nascent [2Fe-2S] cluster intermediate on Isu must already be present for the persulfide sulfur transfer to occur, and its precise cellular origin is still a matter of conjecture [11]. After sulfur transfer, frataxin (Yfh1) leaves the complex, thereby allowing ferredoxin (Yah1) to bind at the Yfh1 overlapping site. This site is characterized by conserved arginine residues and salt bridges. The subsequent Yah1-dependent electron transfer to the sulfur in the nascent cluster converts it to a sulfide as part of an [1Fe-1S] intermediate, which is then converted to a [2Fe-2S] cluster by dimerization of two Isu subunits [12].

Under conditions of frataxin depletion, Fe-S clusters are markedly and globally depleted, including depletion in mitochondrial, cytoplasmic, and nuclear locations. An experiment with human K562 cells, in which frataxin was completely inactivated by CRISPR-based disruption, revealed a virtual complete lack of Fe-S proteins in the knockout [13]. Most likely, in the absence of their Fe-S cluster cofactors, the corresponding Fe-S proteins were destabilized, leading to a myriad of deleterious cellular phenotypes, including impaired respiration, deficient metabolic pathways such as the TCA cycle, altered DNA synthesis and repair, and iron homeostatic perturbation with iron accumulating in mitochondria [13].

Clearly, frataxin/Yfh1 plays an important role in Fe-S cluster assembly and cellular physiology [14,15,16]. Therefore, it was a surprise when a yeast mutant was discovered that could bypass the essentiality and many of the phenotypes of a Δyfh1 deletion strain [17]. In the presence of the M141I allele of the Isu scaffold protein, the growth defects of the Δyfh1 loss-of-function mutant were largely corrected. Fe-S cluster assembly defects were improved, and most cellular Fe-S cluster deficiencies were corrected. In fact, almost all mutant phenotypes were markedly improved, including heme deficiency, respiratory deficiency, and mitochondrial iron accumulation. The mechanism of action of the suppressor Isu was that the M141I allele enhanced persulfide sulfur transfer from Nfs1, either to Isu or perhaps also further downstream to Grx5. The suppression mechanism, thus, was an enhancement of the canonical frataxin function of facilitation of persulfide sulfur transfer [18].

More recently, another frataxin-bypassing protein was described by Mootha and co-workers: METTL17 in humans, which is homologous to Rsm22 in yeast [13]. METTL17 is a mitochondrial protein and is found associated with the small ribosomal subunit in the organelle (mtSSU). The protein was shown to attach to the mitochondrial inner membrane late in the process of ribosome biogenesis, between the SSU ribosomal head and the body. Note that this attachment to the small ribosome subunit occurred prior to the entry of the mRNA into the canal. In elegant structural work, using ribosomes purified from yeast mitochondria, Mootha and co-workers showed that the METTL17 protein initially associated with the ribosome but then “clashed” with mtIF3 (mitochondrial translation initiation factor 3) as it entered the translation channel, implying that the METTL17 protein binding to the complex occurs prior to translation initiation [13]. In this experiment, mtIF3 was expressed in *E. coli*, purified, and then mixed with biochemically purified mitochondrial ribosomes and other fractions from yeast. Biophysical and structural studies also showed METTL17 to have a [4Fe-4S] cluster cofactor [13,19,20]. This [4Fe-4S] cluster might sense changes in the mitochondrial redox environment, possibly inhibiting new mtSSU assembly under oxidative stress conditions [21]. Interestingly, in terms of function, this Fe-S cluster was found to lie downstream of frataxin; i.e., the CRISPR-induced frataxin minus cells lacked the METTL17 Fe-S cluster, resulting in destabilization of the METTL17 protein [13].

Here, working in yeast, we have confirmed many of the results of the Mootha paper [13], with a major difference. In the Mootha paper, the restoration effects of METTL17 overexpression were confined to the mitochondrial Fe-S clusters and respiratory complexes of the edited inactivated frataxin line, leaving the cytoplasmic Fe-S proteins still deficient. The METTL17 overexpression appeared to uncouple growth and bioenergetic effects in frataxin-edited cells, influencing mitochondrial biogenesis of Fe-S clusters at the expense of cytoplasmic Fe-S clusters [13]. On the other hand, working in yeast, we have found a more global suppression of frataxin/Yfh1 depletion phenotypes by the Rsm22 homolog. The Yfh1↓ phenotypic suppression by Rsm22 was not restricted to the mitochondrial synthesis of Fe-S clusters but instead was quite global, with restoration of both mitochondrial and cytoplasmic Fe-S clusters and correction of growth deficits as well. Ferredoxin (Yah1) is an essential component of the mitochondrial iron–sulfur cluster (ISC) machinery. Here, we found that the Yah1 protein level was greatly reduced in Yfh1-depleted mitochondria, but it was restored by Rsm22 overexpression. Strikingly, Yah1 overexpression in Yfh1-depleted cells mimicked many of the salutary effects of Rsm22 overexpression.

## 2. Materials and Methods

### 2.1. Chemicals and Reagents

Chemicals and reagents were mainly purchased from Sigma (St. Louis, MO, USA) or Fisher Scientific (Fair Lawn, NJ, USA). Zymolyase (100T) and raffinose were obtained from US Biological Life Sciences (Salem, MA, USA) and Gold Biotechnology (St. Louis, MO, USA), respectively; ^35^S-labeled cysteine (1000 Ci/mmol) was purchased from Revvity Health Science (Boston, MA, USA). HRP-linked secondary antibodies (donkey anti-rabbit IgG and goat anti-mouse IgG) were purchased from Avantor Science Central (Phillipsburg, NJ, USA) and Fisher Scientific, respectively.

### 2.2. Yeast Strains and Growth Conditions

The native promoter of the *YFH1* gene in the wild-type (WT) strain was substituted with the *GAL1* promoter in the genome [22], resulting in the Gal-Yfh1 strain [17] (Table S1). Both WT and Gal-Yfh1 strains were transformed with a plasmid (pRS425) constructed for constitutive overexpression (↑) of Rsm22 with a C-terminal HA3 tag from the *GPD* promoter, generating GPD-Rsm22/WT and GPD-Rsm22/Gal-Yfh1 strains. The Gal-Yfh1 strain was also transformed with pRS425-GPD-Yah1, generating the strain GPD-Yah1/Gal-Yfh1 for overexpression of ferredoxin. For isolation of mitochondria and/or cytoplasm (see below), cells were grown in selective synthetic complete (SC, with or without the amino acid leucine) containing 2% raffinose and 0.5% dextrose medium [23]. Under these conditions, the *GAL1* promoter in Gal-Yfh1 strains is inactive, resulting in repression (↓) of Yfh1 protein expression. Plasmid-borne overexpression of Rsm22 or Yah1, however, is under the control of *GPD* promoter and is not affected.

### 2.3. Isolation of Mitochondria and Cytoplasm

Mitochondria were isolated/enriched as previously described [24]. Briefly, harvested cells were resuspended in 0.1 M of Tris-sulfate (pH 9.4) and 10 mM of DTT and incubated at 30 °C for 10 min. Following centrifugation, cells were resuspended and treated with Zymolyase 100T (1 mg/g cells) in 20 mM of potassium phosphate (pH 7.4) and 1.2 M of sorbitol at 30 °C for ~45 min. Spheroplasts were collected by centrifugation (4000× *g*, 5 min, 25 °C) and resuspended in ice-cold buffer A (20 mM of HEPES-KOH, pH 7.5; 0.6 M of sorbitol, 0.1% BSA; 1 mM of PMSF; 10 U/mL Trasylol). After Dounce homogenization on ice, samples were centrifuged at 1500× *g* for 5 min at 4 °C. The supernatant was collected, and the pellet was re-homogenized in buffer A and centrifuged. Combined supernatants were centrifuged at 10,000× *g* for 10 min at 4 °C to yield an enriched mitochondrial pellet. Mitochondria were resuspended in HS buffer (20 mM of HEPES-KOH, pH 7.5; 0.6 M of sorbitol) supplemented with 0.1 mg/mL BSA, 10 U/mL Trasylol, and 10% DMSO and stored at −80 °C. The post-mitochondrial supernatant was centrifuged at 153,000× *g* for 45 min at 4 °C, and the resulting supernatant/cytoplasm was stored at −80 °C [24].

### 2.4. Fe-S Cluster Assembly in Isolated Mitochondria

(A) [4Fe-4S] cluster assembly of endogenous aconitase (Aco1): isolated mitochondria (50–400 μg of proteins) were incubated with [^35^S]cysteine (10 μCi), ATP (4 mM), GTP (1 mM), NADH (2 mM), ferrous ascorbate (10 μM), KOAc (40 mM), and Mg(OAc)_2_ (10 mM) in HS buffer (100 μL). Samples were incubated at 30 °C for 10–40 min. Reaction mixtures were diluted with HS buffer and centrifuged (15,000× *g*, 10 min, 4 °C). The resulting mitochondrial pellets (“P”) were resuspended in 50 mM of Tris-HCl (pH 8.0) and 1 mM of PMSF. Membranes were disrupted by freeze/thaw and bath sonication and then centrifuged (15,000× *g*, 30 min, 4 °C). The resulting supernatant fractions containing soluble proteins were subjected to native PAGE and autoradiography, looking for radiolabeling of proteins due to insertion of newly made Fe-^35^S clusters [25].

(B) [2Fe-2S] cluster assembly of imported ferredoxin (Yah1): the full-length ferredoxin precursor protein (pYah1) with a C-terminal His_6_ tag was expressed in *E. coli* BL21 (DE3) cells [25]. Briefly, cells carrying the plasmid pET21b/Yah1-His_6_ were grown at 37 °C in LB (Luria–Bertani) medium containing ampicillin (100 μg/mL) to OD_600_ of ~0.8–1.0. The protein expression was induced with the addition of 1 mM of IPTG (isopropyl-1-thio-β-D-galactopyranoside) for 3 h at 37 °C. The inclusion bodies containing the pYah1 protein were solubilized with 8 M of urea in 50 mM of Tris/HCl, pH 8.0, and centrifuged at 250,000× *g* for 30 min at 25 °C. The pYah1 protein was found to be highly enriched and homogeneous (~80–90%) in the supernatant, and this preparation was directly used for simultaneous mitochondrial protein import and Fe-S cluster assembly. Note that assay conditions for these processes are greatly compatible as both require nucleotides [26]. Briefly, as described above, isolated mitochondria (200 μg of proteins) were supplemented with [^35^S]cysteine, nucleotides (ATP, GTP, NADH), and iron. Following addition of the ferredoxin precursor protein (pYah1; 0.25–1 μg), samples were incubated at 30 °C for 30 min. Radiolabeling of soluble mitochondrial proteins, including endogenous Aco1 and imported Yah1, was analyzed by native PAGE and autoradiography [26].

### 2.5. Cytoplasmic Fe-S Cluster Assembly

In *S. cerevisiae*, the cytoplasmic protein Leu1 (isopropylmalate isomerase) is involved in leucine biosynthesis. The enzyme requires a [4Fe-4S] cluster for its enzymatic activity. The apo-form of Leu1 protein was used as a substrate for cytoplasmic Fe-S cluster assembly [24]. Briefly, *E. coli* BL21 (DE3) cells carrying the plasmids pET21b/Leu1-His_6_ and pBB541-groESL were cultured in LB medium containing spectinomycin (50 μg/mL) and ampicillin (100 μg/mL). Protein expression was induced with IPTG (0.5 mM) for 20 h at 16 °C. Cells were ruptured by sonication and centrifuged. The Leu1-His_6_ protein was purified from the resulting supernatant/soluble fraction using Ni-NTA affinity chromatography. The purified recombinant protein, called Leu1^R^, was found to be predominantly in the apo-form, as judged by its lack of isopropylmalate isomerase activity. The apo-Leu1^R^ protein served as a substrate for cytoplasmic Fe-S cluster assembly as follows.

A typical mitochondria–cytoplasm mixing assay (100 μL) contained mitochondria (200 μg of proteins) isolated from various strains, cytoplasm (200 μg of proteins) isolated from Δleu1 cells, and apo-Leu1^R^ protein (2 μg) in 20 mM of Tris-HCl buffer, pH 7.5, containing 0.6 M of sorbitol, 40 mM of KOAc, and 10 mM of Mg(OAc)_2_. Reaction mixtures were supplemented with unlabeled cysteine (10 μM), nucleotides (4 mM of ATP, 1 mM of GTP, 2 mM of NADH), and ferrous ascorbate (10 μM) and incubated at 30 °C for 30 min. Following centrifugation at 14,000× *g* for 10 min at 4 °C, supernatant fractions containing the cytoplasm were evaluated for reconstituted isopropylmalate isomerase activity. The spectrophotometric assay monitors the change in absorbance at 235 nm due to conversion of 3-isopropylmalate to 2-isopropylmalate over a period of 15 min at 25 °C [24,27,28]. One unit of enzyme activity denotes 18 nmol of product formed/h. Data from independent experiments (n = 3) were plotted using GraphPad Prism 10. The error bars indicate standard deviation.

### 2.6. Miscellaneous

Aconitase activity was measured using either an in-gel assay or a spectrophotometric assay [25,29]. Succinate dehydrogenase activity was assayed by monitoring the reduction of p-iodonitrotetrazolium violet (INT) to its formazan product (INT-formazan) [29,30]. Mitochondrial iron content was chemically determined by dithionite reduction and bathophenanthroline disulfonic acid treatment as described [29]. Mitochondrial membrane potential was evaluated by monitoring fluorescence quenching of the potential-sensitive dye 3,3′-dipropylthiadicarbocyanine iodide [31]. For immunoblotting, mitochondrial proteins were separated by SDS-PAGE, transferred to nitrocellulose membranes, and probed with antibodies.

### 2.7. Data Analysis

For Fe-^35^S cluster loading assays, samples were analyzed by native PAGE followed by autoradiography. Radiolabeled protein bands were quantified via densitometric analysis of scanned autoradiographs using the NIH ImageJ software, version 1.53. Experiments were repeated using biological replicates derived from independent preparations of isolated mitochondria and/or cytoplasm. Cytoplasmic Leu1^R^ Fe–S cluster loading was assessed using spectrophotometric assays (n = 3). Statistical analyses were conducted using GraphPad Prism 10, with data presented as mean ± standard deviation (SD). *p*-values were determined using an unpaired Student’s *t*-test; * *p* < 0.05, ** *p* < 0.01, *** *p* < 0.001, **** *p* < 0.0001, ns, not significant.

## 3. Results

### 3.1. Rsm22 Overexpression Led to Improved Growth of Yfh1-Depleted Cells on a Non-Fermentable Carbon Source

**Figure 1:**
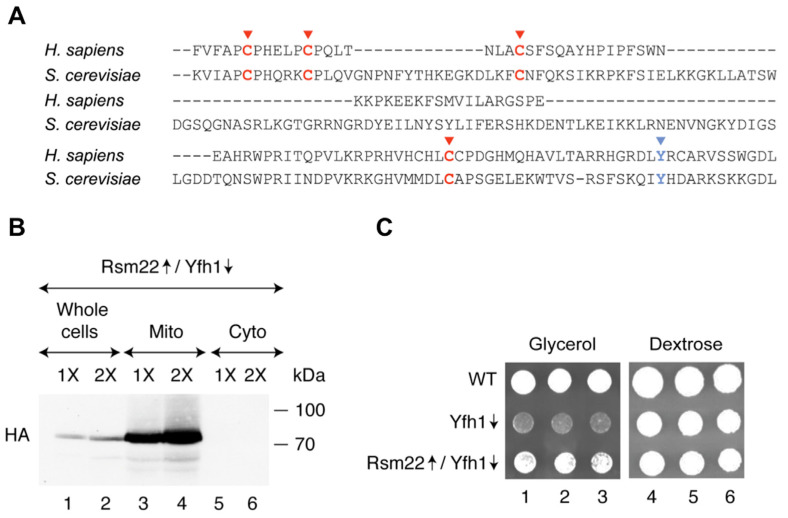
Effects of Rsm22 overexpression in a Yfh1-depleted yeast strain on cell growth. (**A**) Conserved cysteine (**C**) residues involved in Fe-S cluster binding in Rsm22 and its human homolog METTL17 are shown in red [13,19]. The conserved tyrosine (Y) residue of a putative LYR motif is shown in blue. This figure is adapted from Ast et al [13]. (**B**) Mitochondrial localization of overexpressed Rsm22. The native promoter of the *YFH1* gene was replaced with the *GAL1* promoter in the genome [22], generating the Gal-Yfh1 strain (Table S1). This strain was transformed with a plasmid for constitutive overexpression (↑) of Rsm22 with a C-terminal HA3 tag from the *GPD* promoter. The resulting strain was grown in SC medium containing raffinose and dextrose. Under these conditions, the *GAL1* promoter in Gal strains is turned off, and expression of the Yfh1 protein is repressed (↓); this condition is referred to as Rsm22↑/Yfh1↓. Mitochondria and cytoplasm were isolated, and fractions were analyzed by immunoblotting using anti-HA antibodies, looking for Rsm22-HA3 expression/localization. Quantitative analysis of the immunoblot is shown in Figure S2. The original Western blot image can be found in Figure S5. (**C**) Cell growth. The wild-type (WT) and Gal strains were initially grown on an SC plate containing raffinose and galactose. Cells were then transferred to and grown in SC liquid medium containing raffinose and dextrose, harvested, washed, and resuspended in water. Serial 1:5 dilutions of 10^6^ cells were performed, plating the dilutions on YP agar plates with glycerol (left panel) or dextrose (right panel) as the carbon source for growth. The plates were incubated at 30 °C for 3 days, and a photograph was taken.

The yeast Rsm22 is homologous to the human protein METTL17 [32]. Of note, the predicted Cys ligands of the [4Fe-4S] cluster are conserved between the yeast and human proteins (Figure 1A) [13,19]. Also, an LYR/IYH motif is conserved. This motif may mediate Fe-S cluster delivery to recipient target proteins [33]. An Rsm22-HA3 construct (i.e., Rsm22 with three hemagglutinin tags at the C-terminus) was inserted into a plasmid under control of a powerful *GPD* promoter, with the objective of overexpressing this protein. The plasmid was inserted into a Gal-Yfh1 promoter swap strain [17] grown in dextrose medium to repress the genomic Gal-Yfh1 expression, leaving the plasmid-borne GPD-Rsm22 expression unaffected. As can be seen from the immunoblot (Figure 1B), the HA3 tagged Rsm22 was visualized in the whole-cell fractions but not in the cytoplasmic fractions. Further, the HA signal was highly enriched in the mitochondrial fractions, consistent with a mitochondrial localization of endogenous Rsm22 [34]. Our first indication that Rsm22 could act as a global suppressor of Gal-Yfh1↓ was that the Rsm22 plasmid conferred improved growth to the Gal-Yfh1↓ strain grown on a non-fermentable (glycerol) substrate (Figure 1C). Growth of the wild-type (WT) strain on a glycerol plate was robust, whereas the Yfh1↓ strain (repressing conditions) grew very poorly, and Rsm22↑/Gal-Yfh1↓ grew almost like the WT (Figure 1C, left panel). The metabolic changes that made possible this striking growth rescue were unclear at this point.

### 3.2. Rsm22 Overexpression in Yfh1-Depleted Cells Restored Aconitase Protein and Activity

**Figure 2:**
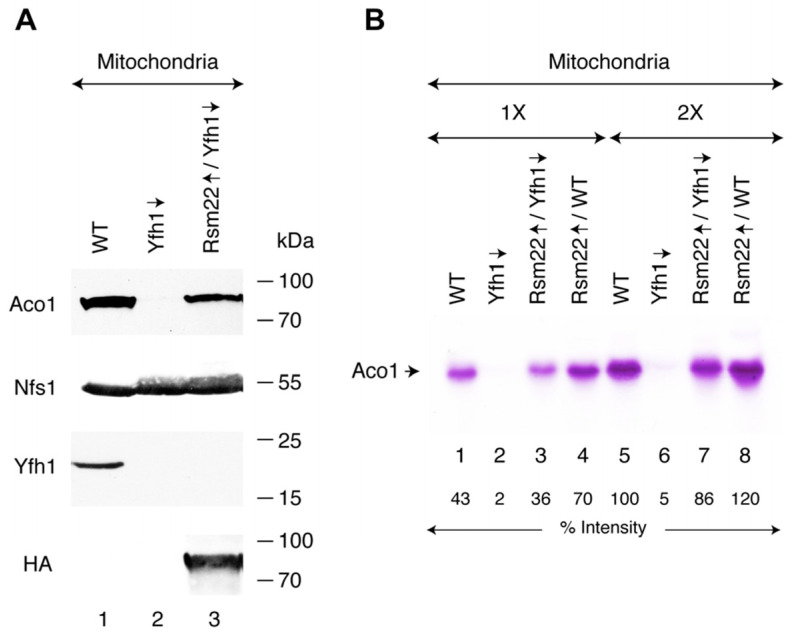
Effects of Rsm22 overexpression in Yfh1-depleted cells on aconitase protein and activity levels. Cells were grown in medium containing raffinose plus dextrose (no galactose, repressing condition for Gal-Yfh1). Mitochondria were isolated from these cells and analyzed. (**A**) Protein levels. Mitochondrial proteins (200 μg) were analyzed by SDS-PAGE, followed by immunoblotting using antibodies against aconitase (Aco1), cysteine desulfurase (Nfs1), Yfh1, and the hemagglutinin (HA) tag for Rsm22. Quantitative data are presented in Figure S2. The original Western blot images can be found in Figure S6, top panel. (**B**) Aconitase activity. Aconitase activity was evaluated by a native in-gel assay [25]. Rsm22↑/WT, Rsm22-HA3 overexpressed in wild-type (WT) cells; 1X = 50 μg of proteins. The intensity of aconitase activity band in wild-type (WT; lane 5) mitochondria was arbitrarily considered as 100%. The original activity gel image can be found in Figure S6, bottom panel.

Aconitase [4Fe-4S] is a TCA cycle enzyme that reversibly converts citrate to isocitrate in mitochondria. Thus, aconitase (Aco1) activity is a good marker for mitochondrial Fe-S cluster assembly status [25]. Mitochondria were isolated from wild-type (WT), Gal-Yfh1 repressed (Yfh1↓), and Rsm22-HA3 overexpressed in Gal-Yfh1 repressed (Rsm22↑/Yfh1↓) cells, and Aco1 protein levels were evaluated by immunoblotting. In WT mitochondria, a strong band indicative of the presence of abundant Aco1 protein was observed, whereas in Yfh1-depleted (Yfh1↓) mitochondria, no such band was seen. Interestingly, overexpression of Rsm22-HA3 in Yfh1↓ mitochondria restored the Aco1 protein level for the most part (Figure 2A). Note that the Nfs1 cysteine desulfurase protein levels were unaffected and served as internal loading controls (Figure 2A). Yfh1 was detectable in WT but not Yfh1-depleted conditions, and Rsm22-HA3 was present only in mitochondria from the strain with the corresponding plasmid. Most likely, aconitase [4Fe-4S] clusters were not generated in the Yfh1-depleted strain, leading to destabilization of the aconitase apoprotein (Figure 2A). A more direct demonstration of the status of the Aco1 Fe-S cluster was obtained by an aconitase in-gel activity assay (Figure 2B). The mitochondria from WT cells showed a clearcut activity band, indicating the presence of enzymatically active aconitase [4Fe-4S] in those mitochondria (Figure 2B, lanes 1 and 5). The mitochondria isolated from Gal-Yfh1↓ cells exhibited no signal at all, consistent with the absence of Fe-S cluster assembly in the frataxin-depleted strain (Figure 2B, lanes 2 and 6). In mitochondria of the strain lacking Yfh1 and overexpressing Rsm22 (i.e., Rsm22↑/Yfh1↓), on the other hand (Figure 2B, lanes 3 and 7), aconitase activity was restored. Interestingly, overexpression of Rsm22 in the WT strain (i.e., Rsm22↑/WT) was associated with an overabundance of aconitase activity, above and beyond the WT levels (Figure 2B, lanes 4 and 8).

### 3.3. Rsm22 Overexpression Restored New Fe-S Cluster Synthesis/Assembly in Isolated Mitochondria Lacking Yfh1

**Figure 3:**
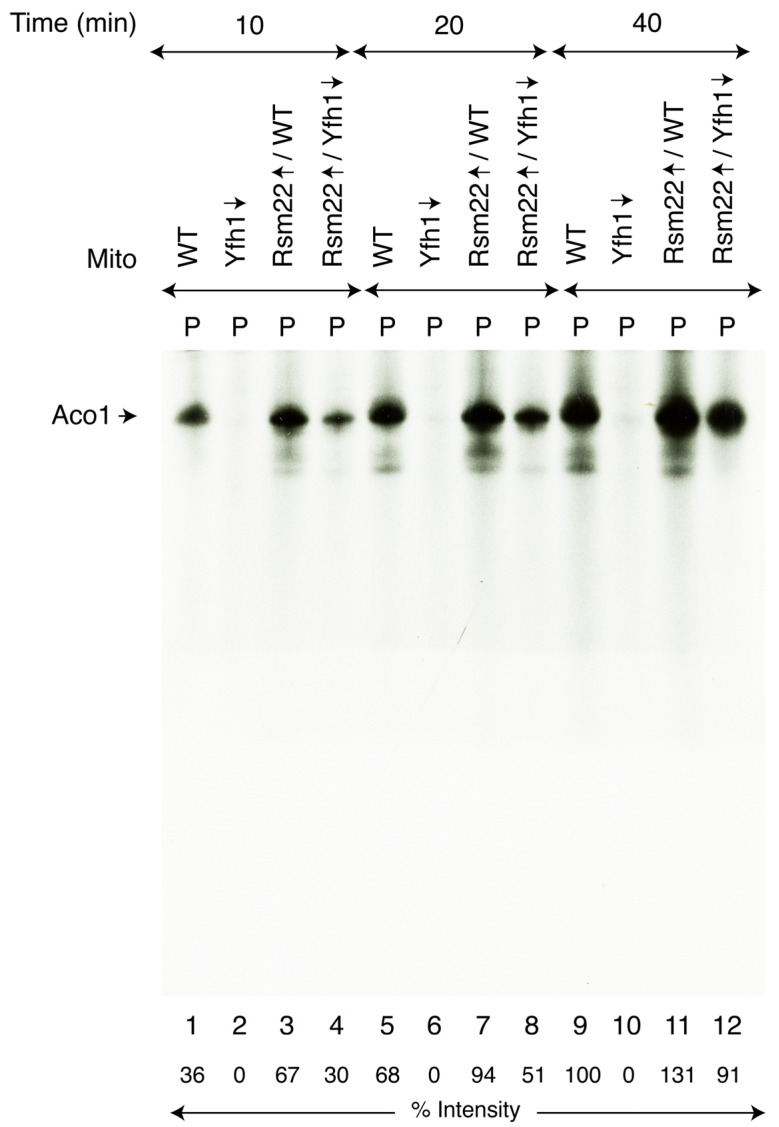
Effects of Rsm22 overexpression on [4Fe-4S] cluster loading of endogenous aconitase in isolated mitochondria. As indicated, various mitochondria (“Mito”; 200 μg of proteins) were supplemented with [^35^S]cysteine, ferrous ascorbate, and nucleotides and incubated at 30 °C for 10–40 min. After centrifugation, the mitochondrial pellets (“P”) were analyzed by native PAGE followed by autoradiography [26]. The relative intensity of Aco1 radiolabeled with [4Fe-4^35^S] clusters is indicated at the bottom of the autoradiograph. The intensity of Aco1 [4Fe-4^35^S] in WT mitochondria at 40 min time point (lane 9) was arbitrarily considered as 100%. The original autoradiograph can be found in Figure S7.

Aconitase protein and activity levels were both restored in Rsm22↑/Yfh1↓ mitochondria, prompting us to determine if these isolated mitochondria can synthesize/assemble [4Fe-4S] clusters for Aco1. For this purpose, we used [^35^S]cysteine as the source of radiolabeled sulfur, and insertion of newly formed Fe-^35^S clusters into endogenous apoAco1 was monitored [25]. Briefly, mitochondria were incubated with [^35^S]cysteine, a nucleotide mixture (ATP, GTP, and NADH), and ferrous ascorbate as the source of iron needed to stimulate new Fe-S cluster assembly in an isolated organelle. Samples were analyzed by native PAGE, followed by autoradiography. The results were parallel in many respects to the activity assays, but here, they reflected new Fe-S cluster assembly. The WT mitochondria exhibited a signal (Figure 3, lanes 1, 5, and 9) migrating near the top of the native gel, reflecting a time course of radiolabeled sulfur incorporation into a newly formed [4Fe-4^35^S] cluster of aconitase. The Yfh1↓ mitochondria displayed no detectable signal (Figure 3, lanes 2, 6, and 10), and the Rsm22↑/Yfh1↓ mitochondria greatly regained the radioactive signal (Figure 3, lanes 4, 8, and 12). Interestingly, Rsm22 overexpression in the WT led to a clearly increased radioactive aconitase signal (Figure 3, lanes 3, 7, and 11), implying that the aconitase enhancement mechanism exerted by Rsm22 overexpression that confers increased aconitase activity in the absence of Yfh1 also functions in the presence of Yfh1.

Similarly, biosynthesis of mitochondrial [2Fe-2S] cluster proteins such as ferredoxin (Yah1) was rescued. Like aconitase, ferredoxin is another mitochondrial matrix protein. However, unlike Aco1, Yah1 is much less abundant. We therefore tested Fe-^35^S cluster loading of ferredoxin after importing the corresponding precursor protein into isolated mitochondria [25]. The ferredoxin precursor protein (pYah1) was expressed in bacteria, and inclusion bodies containing the protein were solubilized with urea to obtain apo-pYah1 for mitochondrial import and subsequent Fe-S cluster assembly. Briefly, mitochondria (WT, Yfh1↓, or Rsm22↑/Yfh1↓) were incubated with pYah1 in the presence of added [^35^S]cysteine, nucleotides, and iron. Mitochondria were reisolated by centrifugation, and the samples were analyzed by native PAGE, followed by autoradiography. Upon import into the mitochondrial matrix, the pYah1 precursor protein was converted to the corresponding mature form, which then served as a substrate for [2Fe-2S] cluster assembly by the mitochondrial ISC machinery. Notably, the conditions for mitochondrial protein import and Fe-S cluster loading are highly compatible, as both processes require nucleotides [26].

**Figure 4:**
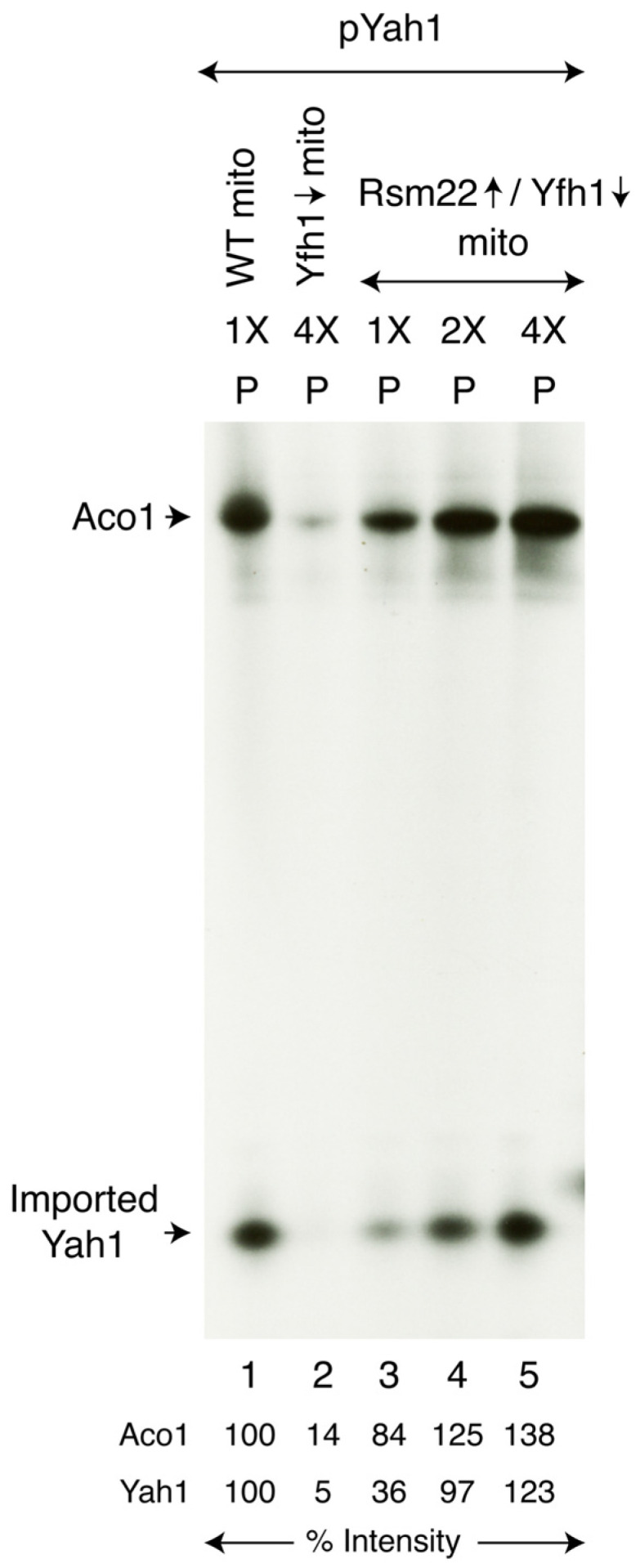
Effects of Rsm22 overexpression on [2Fe-2S] cluster loading of newly imported ferredoxin in Yfh1-depleted mitochondria. Various mitochondria (“mito”; 200 μg of proteins) were incubated with the pYah1 precursor protein (1X = 0.25 μg) in the presence of [^35^S]cysteine, ferrous ascorbate, and nucleotides at 30 °C for 30 min. After centrifugation, mitochondrial pellets (“P”) were analyzed by native PAGE, followed by autoradiography. The original autoradiograph can be found in Figure S8.

Imported ferredoxin (Yah1) was loaded with newly formed Fe-^35^S clusters in WT but not in Yfh1↓ mitochondria (Figure 4, lanes 1 and 2). In mitochondria isolated from the suppressed strain (Rsm22↑/Yfh1↓), Yah1 loading with a [2Fe-2^35^S] cluster was clearly visible above the background, and the signal intensity increased with increasing mitochondrial load (Figure 4, lanes 3–5). Note that the signal on Yah1 was dependent on in vitro mitochondrial import of a precursor protein. The results are therefore complicated because import efficiency of the precursor protein was decreased in the Yfh1↓ mitochondria due to low membrane potential (see below). Both mitochondrial import efficiency and Fe-S loading capability were restored to some extent by Rsm22 overexpression. In summary, Fe-S cluster loading of imported Yah1 was active in WT mitochondria, absent in Yfh1↓ mitochondria, and partially restored in Rsm22↑/Yfh1↓ mitochondria. This experiment (Figure 4) represents another demonstration of the suppressive effects of Rsm22 on Yfh1↓ phenotypes.

### 3.4. Overexpression of Rsm22 Rescued Other Iron Proteins, Restored Iron Homeostasis, and Conferred Membrane Potential in Mitochondria Lacking Yfh1

**Figure 5:**
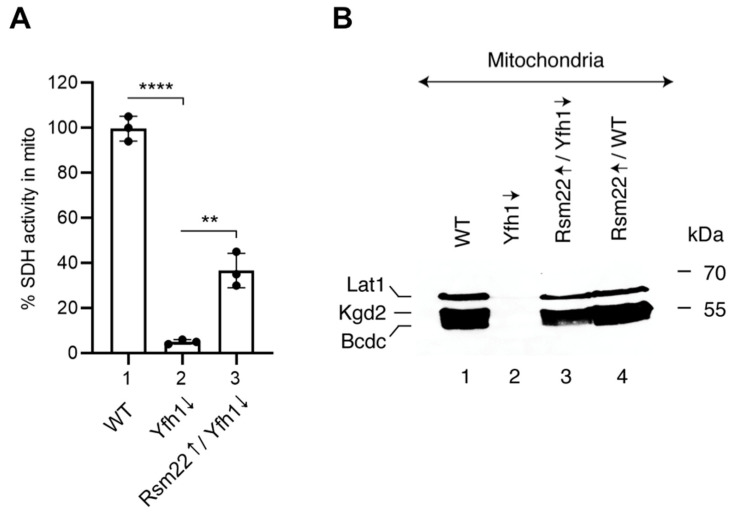
Effects of Rsm22 overexpression on other proteins in Yfh1-depleted mitochondria. (**A**) SDH activity. Succinate dehydrogenase activity in various mitochondria (100 μg of proteins) was measured by a spectrophotometric assay [29]. SDH activity in wild-type (WT) mitochondria was arbitrarily considered as 100%. (**B**) Immunoblot. Mitochondrial proteins (200 μg) were analyzed by SDS-PAGE, followed by immunoblotting using antibodies against lipoic acid (LA) [35,36]. Quantitative data are presented in Figure S2. The original Western blot image can be found in Figure S9. *p*-values were determined using an unpaired Student’s *t*-test; ** *p* < 0.01, **** *p* < 0.0001.

The set of WT, Yfh1↓, and Rsm22↑/Yfh1↓ mitochondria was analyzed for the steady state status of selected Fe-S proteins. Succinate dehydrogenase, a [2Fe-2S] protein that constitutes complex II of the mitochondrial respiratory chain, exhibited strong activity in WT mitochondria and negligible activity in Yfh1↓ mitochondria (less than 5%) but recovered to a ~35% level in Rsm22↑/Yfh1↓ mitochondria, indicating effective, albeit partial, rescue of the mutant phenotype (Figure 5A). Information on proteins containing lipoic acid (LA) in these mitochondria was gleaned by immunoblotting. These proteins are dependent on Fe-S cluster formation in mitochondria for their biogenesis and stability. An immunoblot using anti-LA antibodies (Figure 5B) revealed three distinct bands in WT mitochondria corresponding to the E2 subunits of mitochondrial dehydrogenase complexes: Lat1 of the pyruvate dehydrogenase complex, Kgd2 of the α-ketoglutarate dehydrogenase complex, and Bcdc of the branched-chain α-keto acid dehydrogenase complex [35]. A fourth lipoic-acid-containing protein Gcv3, the H subunit of the glycine cleavage system, was not seen, perhaps due to its small size. What is clear is that the signals for lipoic-acid-binding subunits in WT mitochondria (Figure 5B, lane 1) were completely absent in the Yfh1↓ mitochondria (Figure 5B, lane 2) but were restored almost quantitatively in the Rsm22↑/Yfh1↓ mitochondria (Figure 5B, lane 3). The effects likely reflect the status of lipoic acid synthase, a protein with two iron–sulfur clusters, one [4Fe-4S] cluster which serves as a radical SAM donor and a second [4Fe-4S] cluster which serves as a sulfur donor for formation of the LA cofactor [6].

**Figure 6:**
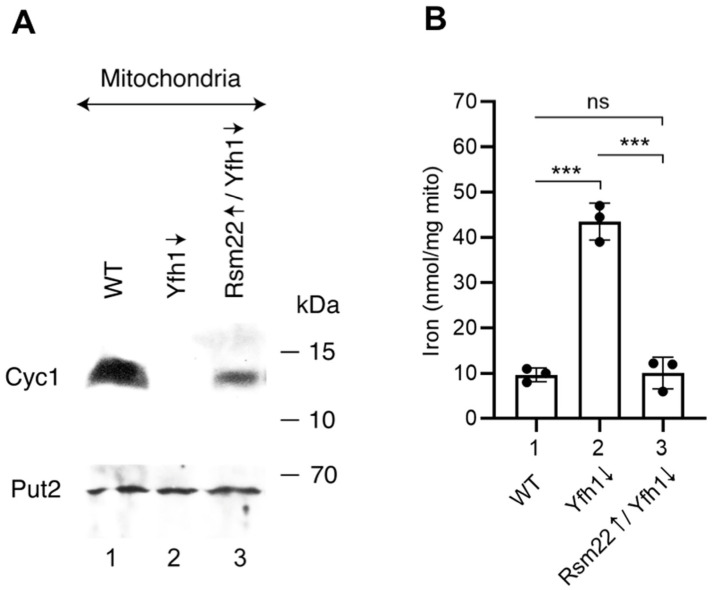
Effects of Rsm22 overexpression on cytochrome *c* and iron levels in Yfh1-depleted mitochondria. (**A**) Immunoblot. Mitochondrial proteins (200 μg) were analyzed by SDS-PAGE, followed by immunoblotting using antibodies against Cyc1 (top panel) or Put2 (bottom panel). Quantitative data are presented in Figure S2. The original Western blot images can be found in Figure S10. (**B**) Mitochondrial iron. Various mitochondria (500 μg of proteins) were treated with 1% SDS, 1 mM of dithionite, and 1 mM of BPS (bathophenanthroline disulfonic acid) at 37 °C for 60 min. Samples were centrifuged at 14,000× *g* for 10 min at 25 °C, and absorbance of the supernatant was measured at 515 nm [29]. *** *p* < 0.001, ns, not significant.

Cytochrome *c* transfers electrons from the respiratory complex III to complex IV as part of the electron transport chain in the mitochondrial inner membrane. It is a non-Fe-S heme protein, whose stability is dependent on the presence of the heme *c* cofactor [37]. In Yfh1↓ mitochondria, cytochrome *c* was undetectable by immunoblotting (Figure 6A, compare lanes 1 and 2), but levels were partially restored in Rsm22↑/Yfh1↓ mitochondria (Figure 6A, lane 3). Cytochrome *c* deficiency in Yfh1↓ cells probably results from cellular heme deficiency. This, in turn, may be due to a deficiency in lipoic acid. The lipoic acid deficiency causes a defect in the lipoic-acid-dependent TCA cycle enzyme alpha-ketoglutarate dehydrogenase, leading to succinyl CoA deficiency. Likewise, delta-aminolevulinic acid (ALA), a critical intermediate in the heme synthesis, is also deficient [38,39]. Rsm22 expression, by restoring lipoic acid synthesis in the absence of Yfh1, restored heme synthesis and raised cytochrome *c* levels.

Mitochondrial iron accumulation of Gal-Yfh1↓ was reversed by overexpression of Rsm22; i.e., mitochondrial iron levels were returned to normal (Figure 6B). In situations of frataxin deficiency, a distinctive iron homeostatic phenotype ensues [29]. Iron floods into the cell due to activation of the Aft1/2 transcription factor, which turns on high-affinity cellular iron transporters. A regulatory [2Fe-2S] cluster on the Aft1/2 proteins is not formed, leading to binding of the apoprotein to cognate DNA target sites and activation of the iron regulon, thereby driving cellular iron uptake [40]. In addition to increased cellular iron uptake, these misregulated cells accumulate iron in mitochondria. Mitochondrial iron may oxidize and aggregate, forming insoluble nanoparticles [41]. We measured iron in WT mitochondria using a combination of SDS to solubilize the iron and dithionate to reduce the iron to the ferrous valence state. The solubilized/reduced samples were then assessed in the presence of bathophenanthroline as a quantitative colorimetric indicator for the amount of iron present [29]. WT mitochondria showed a basal level of iron (~10 nmole/mg of proteins), whereas Yfh1↓ mitochondria exhibited iron accumulation (~44 nmole/mg of proteins). Rsm22 overexpressing mitochondria in the Yfh1↓ strain showed correction of mitochondrial iron levels back to the WT basal levels (Figure 6B). Again, the effect of Rsm22 overexpression was to return the Yfh1↓ mutant phenotype back to normal, conferring suppression of the frataxin minus phenotypes.

The phenotypes of Yfh1↓ cells are very diverse, perhaps related to the global Fe-S cluster deficiency engendered by lack of frataxin, a core component of the Fe-S cluster assembly complex. Mitochondrial membrane potential depends on the intactness of various aspects of mitochondrial physiology, ranging from NADH production to respiratory complexes to proton pumping, all of which utilize Fe-S clusters for their functions. We wondered if the deficiency in frataxin was reflected in effects on the mitochondrial membrane potential. The membrane potential of isolated mitochondria was measured using the DiSC3(5) fluorescent dye. This dye is accumulated/concentrated in mitochondria in a membrane-potential-dependent manner, thereby quenching the signal. WT mitochondria exposed to the dye produced ~2000 units of signal (Figure S1A). The Yfh1↓ mutant mitochondria showed decreased membrane potential (increased DiSC3(5) fluorescence) of ~5000 units, and Rsm22 overexpression in this context conferred complete recovery of the membrane potential to WT levels, decreasing the signal to ~1200 units (Figure S1A). The recovered membrane potential indicates restored respiratory complex function and corresponding proton pumping activity even though frataxin is still absent. In the presence of valinomycin, the membrane potential across the mitochondrial inner membrane was dissipated, and more dye was released, producing a higher fluorescent signal of 10,000–12,000 units (Figure S1B). Thus, Rsm22 confers a global correction of the mutant phenotypes on the Yfh1↓ strain, including correction of the deficient mitochondrial membrane potential.

### 3.5. Yfh1↓ Mitochondria with Overexpressed Rsm22 Regained Capability to Promote Cytoplasmic Fe-S Cluster Assembly

In eukaryotes, Fe-S cluster biogenesis is compartmentalized, requiring a mitochondrial iron–sulfur cluster (ISC) machinery and a cytosolic iron–sulfur protein assembly (CIA) machinery. The mitochondrial ISC lies upstream of the CIA machinery [26,42], and thus, deficiency in any of the core components of the ISC machinery leads to impaired cytoplasmic Fe-S cluster biosynthesis. Isolated mitochondria by themselves can synthesize new Fe-S clusters (e.g., Figure 3 and Figure 4). In contrast, isolated cytoplasm by itself cannot make Fe-S clusters. Addition of mitochondria to the cytoplasm, however, allows cytoplasmic Fe-S cluster synthesis [26]. A mixture of mitochondria and cytoplasm is required for cytoplasmic Fe-S cluster assembly, as both make important contributions. Mechanistically, mitochondria synthesize and export a critical intermediate, called (Fe-S)_int_, to the cytoplasm for Fe-S cluster assembly in that compartment [26,42]. We have developed isolated mitochondria–isolated cytoplasm mixing assays to study the roles of a number of ISC (and CIA) components involved in cytoplasmic Fe-S cluster synthesis [24,26]. Here, we used these assays to determine the effects of Rsm22 overexpression on cytoplasmic Fe-S cluster assembly.

**Figure 7:**
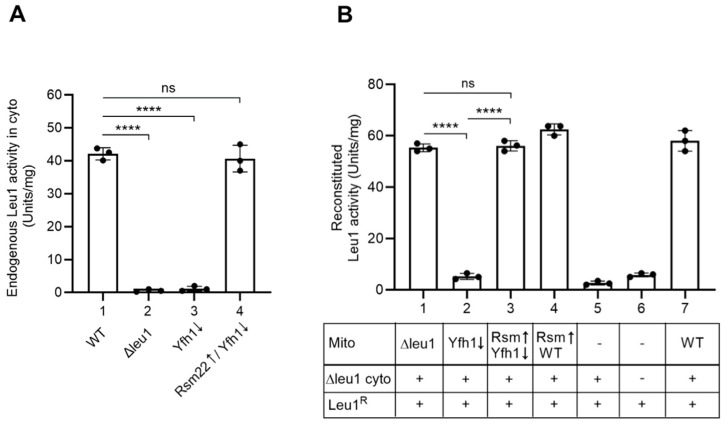
Effects of Rsm22 overexpression in Yfh1-depleted mitochondria on cytoplasmic Fe-S cluster assembly. (**A**) Endogenous Leu1 [4Fe-4S] enzyme activity in isolated cytoplasm. Cytoplasm was isolated from wild-type (WT), Gal-Yfh1 repressed (Yfh1↓), Rsm22 overexpressed in Gal-Yfh1 repressed (Rsm22↑/Yfh1↓), and *LEU1* gene deleted (Δleu1) strains. Endogenous Leu1 isopropylmalate isomerase activity was measured in these cytoplasmic samples containing 200 μg of proteins [24]. (**B**) Reconstitution of bacterial expressed and purified apo-Leu1^R^ protein in isolated Δleu1 cytoplasm. As indicated, isolated mitochondria (200 μg of proteins) were mixed with isolated Δleu1 cytoplasm (200 μg of proteins) and apo-Leu1^R^ protein (2 μg). All reaction mixtures were supplemented with unlabeled cysteine (10 μM), nucleotides (4 mM of ATP, 1 mM of GTP, 2 mM of NADH), and ferrous ascorbate (10 μM) and incubated at 30 °C for 30 min. After removal of mitochondria by centrifugation, the resulting cytoplasm/supernatant fractions were assayed for reconstituted Leu1^R^ isopropylmalate isomerase activity as in (**A**) above [24]. **** *p* < 0.0001, ns, not significant.

The Leu1 isopropylmalate isomerase was chosen as the cytoplasmic Fe-S protein marker. The enzyme requires a [4Fe-4S] cluster for activity and is responsible for isomerizing isopropylmalate from alpha to beta isoforms as part of the leucine biosynthesis pathway [24]. Leu1 is an excellent indicator of cytoplasmic Fe-S cluster status. The endogenous Leu1 activity in various cytoplasm isolates was as expected, with WT showing high activity of ~41 units/mg, and Δleu1 and Yfh1↓ showing negligible activity. Strikingly, however, cytoplasm isolated from Rsm22↑/Yfh1↓ cells showed almost completely restored activity of ~40 units/mg (Figure 7A). The overall protein pattern of various cytoplasm was found to be very similar, serving as a loading control (Figure S3). To further substantiate the stimulatory effects of Rsm22 overexpression on Leu1 enzyme activation more directly, we took advantage of the mitochondria–cytoplasm mixing assays as follows.

Mitochondria were isolated from WT and various mutant strains. Cytoplasm was generally derived from a Δleu1 strain so that there was no background signal. For the assay, bacterial expressed and purified apol-Leu1^R^ was added to the mixture of mitochondria and cytoplasm, and after incubation, the formation of new Leu1 holoprotein was assessed by a biochemical spectrophotometric assay for newly reconstituted isopropylmalate isomerase activity [24]. Using this assay, WT mito/Δleu1 cyto combination produced a robust signal of about 60 units/mg of Leu1 activity (Figure 7B, bar 7), whereas Yfh1↓ mito/∆leu1 cyto combination exhibited a very low signal of 2 units/mg of Leu1 activity (Figure 7B, bar 2). Strikingly, Rsm22↑ expression in Yfh1↓ mitochondria with overexpressed Rsm22 (i.e., Rsm22↑/Yfh1↓) led to complete restoration of Leu1 activity to ~60 units/mg (Figure 7B, bar 3). Rsm22↑/WT mito mixed with Δleu1 cyto appeared very much like the WT/Δleu1 combination (Figure 7B, bar 4), and mixtures without mitochondria exhibited negligible activity (Figure 7B, bars 5 and 6).

These results show that Yfh1↓ mitochondria are associated with defects in cytoplasmic [4Fe-4S] cluster synthesis that are almost completely recovered by overexpression of Rsm22 in the Yfh1↓ mitochondria. Rsm22-overexpressing Yfh1↓ mitochondria can provide signals to the cytoplasm, probably via synthesis and export of the (Fe-S)_int_ intermediate. The exported intermediate stimulates cytoplasmic production of the Fe-S clusters that are then incorporated into apoLeu1 to make the holo and active enzyme. In summary, Rsm22-mediated Yfh1 suppression appears quite global, affecting both mitochondrial and cytoplasmic Fe-S cluster functions. The linkage of mitochondrial production of a key intermediate (Fe-S)_int_ and cytoplasmic Fe-S cluster assembly activity has been detailed elsewhere [6].

### 3.6. A Possible Suppression Mechanism

**Figure 8:**
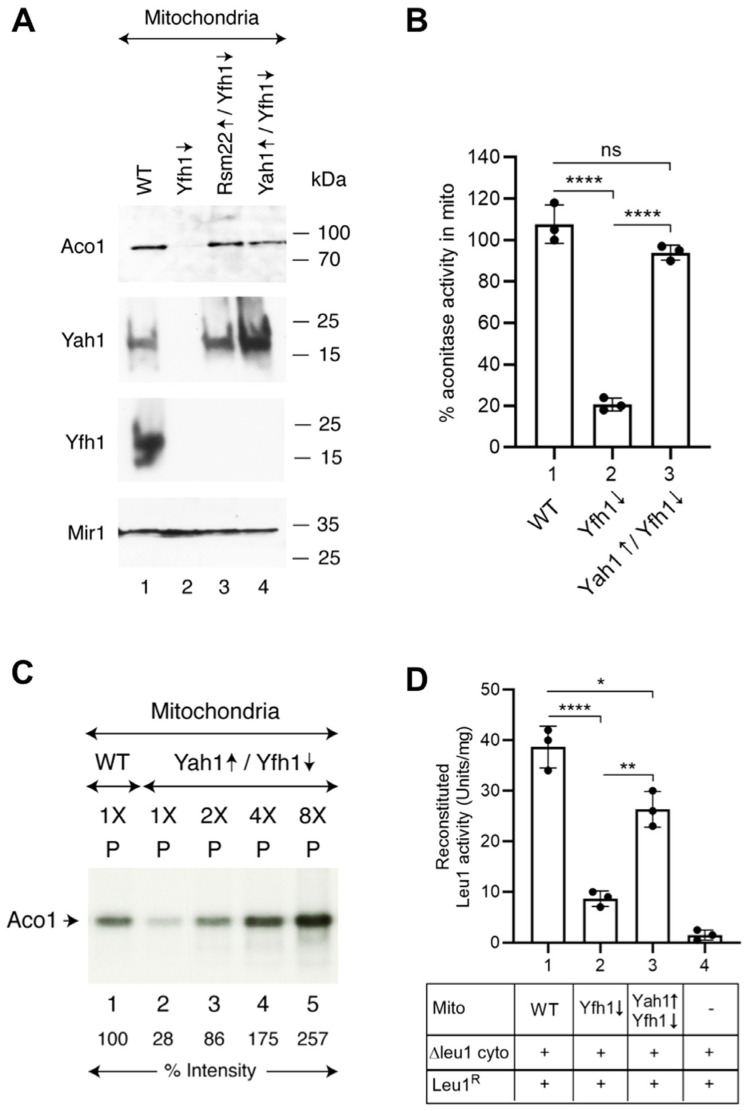
Effects of ferredoxin (Yah1) overexpression in Yfh1-depleted cells on mitochondrial and cytoplasmic Fe-S protein biogenesis. The Gal-Yfh1 strain was transformed with a plasmid for constitutive overexpression (↑) of full-length ferredoxin from the *GPD* promoter. All cells were grown in SC medium containing raffinose and dextrose, and mitochondria were isolated. Yah1↑/Yfh1↓ and ferredoxin overexpressed in Gal-Yfh1 repressed mitochondria. (**A**) Protein levels. Mitochondrial proteins were analyzed by SDS-PAGE, followed by immunoblotting using antibodies against Aco1, Yah1, and Yfh1. Immunoblotting using anti-Mir1 antibodies served as internal loading control. Quantitative data are presented in Figure S2. The original Western blot images can be found in Figure S11. (**B**) Aconitase activity. Isolated mitochondria (200 μg of proteins) were lysed, and aconitase activity was assessed by a spectrophotometric assay [29]. (**C**) Fe-^35^S cluster loading of aconitase. Isolated mitochondria (WT or Yah1↑/Yfh1↓; 1X = 50 μg of proteins) were incubated with [^35^S]cysteine (10 μCi), nucleotides (4 mM of ATP, 1 mM of GTP, 2 mM of NADH), and ferrous ascorbate (10 μM) at 30 °C for 30 min. Reaction mixtures were diluted with isotonic buffer and centrifuged, and the resulting mitochondrial pellets (“P”) were analyzed by native PAGE followed by autoradiography [26]. The relative intensity of radiolabeled Aco1 is indicated. The intensity of Aco1 [4Fe-4^35^S] in WT mitochondria (lane 1) was arbitrarily considered as 100%. The original autoradiograph can be found in Figure S12. (**D**) Leu1^R^ reconstitution in isolated Δleu1 cytoplasm. As indicated, mitochondria (200 μg of proteins) were mixed with Δleu1 cytoplasm (200 μg of proteins) and apo-Leu1^R^ protein (2 μg). Reaction mixtures were supplemented with unlabeled cysteine, nucleotides, and iron. After incubation at 30 °C for 30 min, Leu1^R^ enzyme activation was measured as in Figure 7B [24]. * *p* < 0.05, ** *p* < 0.01, **** *p* < 0.0001, ns, not significant.

We wondered how Rsm22↑ might suppress Yfh1↓ phenotypes. The suppression was global, affecting virtually all Yfh1↓ phenotypes, from growth deficiencies on non-fermentable media to mitochondrial status, including membrane potential, Fe-S clusters in mitochondria, Fe-S clusters in the cytoplasm, and iron homeostatic phenotypes. We considered that Rsm22 might alter/affect the core mitochondrial ISC machinery. This is the way that the Isu1 M141I allele bypasses frataxin loss, i.e., by altering persulfide sulfur transfer activity from Nfs1 early on in mitochondrial Fe-S cluster synthesis [17]. Here, in this case, we wondered whether Yah1 was involved, as Yah1 is also a core Fe-S cluster assembly component, interacting with Nfs1 and acting early on in the Fe-S assembly scheme. After all, Yah1 protein, a [2Fe-2S] cluster ferredoxin, was absent in Yfh1↓ but restored in Rsm22↑/Yfh1↓ mitochondria (Figure 8A). The Yah1 ferredoxin acts as part of the Fe-S cluster assembly process, immediately subsequent to the action of Yfh1, likely intervening following persulfide sulfur transfer to Isu, to donate an electron to the nascent Fe-S cluster [12]. In fact, Yah1 competes with Yfh1 for binding to the Nfs1 core, interacting with a conserved Arg+ patch on Nfs1. Yah1 in WT binds to the Nfs1/Isd11/Acp1/Isu core after the departure of Yfh1, thereby moving close to the Isu scaffold and donating an electron to reduce sulfur to sulfide in the nascent [2Fe-2S] cluster [12]. Could it be that Yah1 binding to Nfs1 and Nfs1/Isd11/Acp1/Isu core in the absence of Yfh1 actually supplants some of its functions needed for Fe-S cluster biosynthesis? This possibility was tested as follows.

The *YAH1* open reading frame was placed under control of a powerful *GPD* promoter, the protein was overexpressed (Yah1↑) from a high copy-number plasmid in the Yfh1↓ strain, and mitochondria were isolated. Four different mitochondria were examined (Figure 8A): WT, Yfh1↓, Rsm22↑/Yfh1↓, and Yah1↑/Yfh1↓. WT mitochondria demonstrated the presence of aconitase protein, and Yfh1↓ mitochondria had no detectable aconitase protein. Both Rsm22↑/Yfh1↓ and Yah1↑/Yfh1↓ mitochondria showed restoration of aconitase protein levels (Figure 8A). Activity assays for aconitase confirmed that WT had 100% activity, Yfh1↓ less than 20%, and Yah1↑/Yfh1↓ exhibited ~90% activity (Figure 8B). Rsm22 overexpression thus corrected many Fe-S cluster mutant phenotypes in Yfh1↓, as did Yah1. Rsm22 overexpression restored Yah1 expression in Yfh1↓, and Yah1 overexpression in Yfh1↓ restored aconitase protein and activity. Loading of endogenous Aco1 with newly made Fe-^35^S clusters in isolated Yah1↑/Yfh1↓ mitochondria was also restored, and the radiolabeled Aco1 signal increased with increasing concentrations of mitochondria used in the assay (Figure 8C). Rsm22 might therefore reestablish Fe-S cluster biosynthesis by restoring Yah1 protein expression and activity, which could act on the core Nfs1/Isd11/Acp1/Isu complex to facilitate persulfide sulfur transfer and reduction, i.e., similar to frataxin.

To further validate this hypothesis, we tested if Yah1 overexpression, like in the case of Rsm22 overexpression, can also restore the ability of Yfh1↓ mitochondria to promote cytoplasmic Fe-S cluster assembly using the spectrophotometric assay for Leu1^R^ enzyme activation. Briefly, apol-Leu1^R^ was added to a mixture of mitochondria (WT, Yfh1↓, or Yah1↑/Yfh1↓) and cytoplasm (Δleu1). After incubation, the Leu1 holoprotein formation was assessed by the appearance of isopropylmalate isomerase activity. Whereas Yfh1↓ mito/∆leu1 cyto exhibited a low signal of ~5 units/mg, a significantly enhanced signal of 25 units/mg was observed for the Yah1↑/Yfh1↓ mito with Δleu1 cyto combination (Figure 8D). Together, these results imply that the salutary effects of Rsm22 overexpression in Yfh1↓ mitochondria could be mediated, directly or indirectly, through restoration of Yah1 protein and activity.

## 4. Discussion

Soon after the discovery of the human frataxin homolog in yeast (called Yfh1) [43], it was recognized that frataxin deletion in yeast was deleterious. These deletion effects were often sublethal [44], as distinguished from the effects of, for example, deletion of Nfs1. Nfs1, encoding the cysteine desulfurase enzyme at the core of the Fe-S cluster assembly complex, was found to be absolutely essential, and a deletion could not be bypassed or suppressed [29]. On the other hand, in the face of frataxin deletion, extragenic suppressor mutations occurred with high frequency, mitigated only in the setting of hypoxic growth conditions [17]. One of these suppressor mutants was further analyzed and found to carry a point mutation in the scaffold protein gene Isu1, M141I. Remarkably, the effect of the mutation was to bypass or suppress most of the Δyfh1 phenotypes—including Fe-S cluster deficiency in multiple target proteins. In addition, growth was corrected, cytochromes and heme were restored, and mitochondrial iron accumulation was reversed. This bypass phenomenon could be understood in terms of the activity of a mutant allele of Isu1, which was able to accelerate persulfide sulfur transfer in the absence of frataxin [18]. Thus, the suppressor Isu influenced core functions of the Fe-S cluster assembly machinery, accelerating what was the critical frataxin-dependent step (albeit in the absence of frataxin).

**Figure 9:**
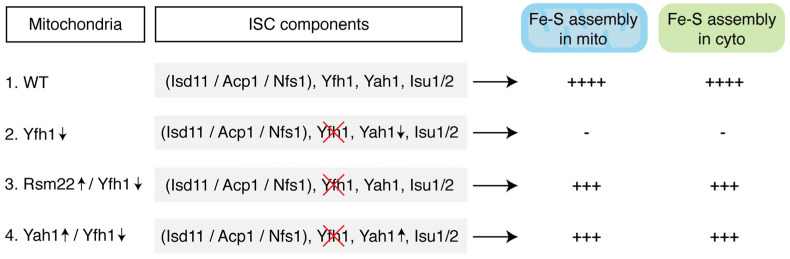
Model for salutary effects of Rsm22 overexpression on Yfh1 depletion phenotypes. 1. Wild-type (WT) mitochondria: iron–sulfur cluster (ISC) assembly complex is made up of the core components Isd11, Acp1, Nfs1, and Isu1/2, with accessory components, such as Yfh1 and Yah1, that bind to a conserved arginine patch on Nfs1 [12]. The complex functions in synthesis of [2Fe-2S] clusters on Isu1/2. Isolated WT mitochondria can efficiently promote Fe-S cluster assembly in isolated WT cytoplasm. 2. Yfh1↓ mitochondria: Yfh1 is largely absent, Fe-S clusters are destabilized including ferredoxin (Yah1), leading to deficiency in Fe-S clusters in mitochondria and cytoplasm. Isolated Yfh1↓ mitochondria cannot promote Fe-S cluster assembly in isolated WT cytoplasm. 3. Rsm22↑/Yfh1↓ mitochondria: Rsm22 overexpression in mitochondria restores/stabilizes Yah1 protein/activity even in the absence of Yfh1, and mitochondrial and cytoplasmic Fe-S enzyme activities are largely restored. Unlike Yfh1↓ mitochondria, Rsm22↑/Yfh1↓ mitochondria can promote Fe-S cluster assembly in isolated WT cytoplasm. 4. Yah1↑/Yfh1↓ mitochondria: Yah1 overexpression in Yfh1↓ mitochondria mimics the salutary effects of Rsm22 overexpression in Yfh1↓, in terms of Fe-S cluster assembly in mitochondria and cytoplasm. For the biosynthetic processes tested, the efficiency of WT mitochondria was arbitrarily considered 100% as indicated by four pluses (“++++”). Three pluses (“+++”), activity ranging from ~30 to 90%; minus sign (“-”), no significant activity; red cross (“X”), Yfh1 protein absent.

Here we describe a similar frataxin-bypass phenomenon, in this case, mediated by Rsm22, which is homologous to the human METLL17. The results are summarized in Figure 9. Rsm22 overexpression in the setting of Yfh1 depletion largely and generally returned the mutant phenotypes towards normal—if not completely, then in that direction. Specifically, growth of the Yfh1↓ strain on a non-fermentable carbon source such as glycerol medium was restored (Figure 1). Aconitase activity came back (Figure 2B), and aconitase Fe-S cluster loading, assessed by a radioactive in organelle mitochondrial assay, was partially restored (Figure 3). Other mutant phenotypes were restored as well, including lipoic acid synthesis (Figure 5B), dependent on biosynthesis of the Fe-S cluster protein lipoic acid synthase. Iron accumulation in mitochondria in the form of nanoparticle aggregates was reversed (Figure 6B). Importantly, the suppression extended to involve cytoplasmic Fe-S cluster assembly (Figure 7).

As we know, cytoplasmic Fe-S cluster assembly lies downstream of mitochondrial Fe-S cluster biosynthesis because of its dependence on (Fe-S)_int_, a still poorly characterized mitochondrial synthesized intermediate probably containing iron and sulfur [5,6]. Mitochondria, and more specifically, the mitochondrial ISC machinery, makes and exports (Fe-S)_int_, which is utilized in the cytoplasm to mediate Fe-S cluster assembly in that compartment. In Yfh1↓ mutant cells, activity of the Leu1 isopropylmalate isomerase, a cytoplasmic [4Fe-4S] protein, was virtually absent, whereas in the Rsm22 overexpressing strain still lacking expression of Yfh1, Leu1 activity was completely restored in vivo (Figure 7A). These findings were independently validated by a newly developed biochemical mixing assay in which separate mitochondrial and cytoplasmic components were combined, and purified Leu1 apoprotein was provided. The source of the mitochondria was a strain with overexpressed Rsm22 in the context of Yfh1↓, whereas the source of the cytoplasm was a Δleu1 mutant. The results of the mixing showed full restoration of cytoplasmic Fe-S cluster synthesis capability on Leu1 by the Rsm22↑/Yfh1↓ mitochondria (Figure 7B). We should note that, in this regard, our results are at variance with those of Mootha and co-workers working on METTL17 in human cells [13]. They observed a dissociation of mitochondrial and cytoplasmic Fe-S cluster restoration due to METTL17 overexpression, in that only mitochondrial clusters such as respiratory complex Fe-S clusters were restored, whereas cytoplasmic Fe-S clusters such as PolD were not recovered [13]. The experiments by Mootha and co-workers were performed in human K562 cells edited for frataxin, whereas our experiments were performed in the yeast *S. cerevisiae*, depleted for Yfh1 by promoter swap and repression. The discrepancy between human and yeast results remains to be further clarified.

We wondered about the mechanism of the Rsm22 bypass of frataxin loss. Frataxin function is now known to involve regulation of the rate and efficiency of persulfide sulfur transfer from Nfs1 to downstream recipients such as Isu1/2 in an early step of Fe-S cluster assembly in mitochondria [9]. Therefore, we thought perhaps Rsm22 might enhance this rate or the efficiency of persulfide sulfur transfer in the absence of frataxin. Extensive experimentation was performed with isolated mitochondria to visualize and characterize the Nfs1 persulfide in WT, Yfh1↓, and Rsm22↑/Yfh1↓ mitochondria. The results revealed that the Nfs1-bound persulfide was increased in the absence of frataxin, perhaps because persulfide sulfur transfer was impaired. However, the Rsm22↑/Yfh1↓ mitochondria displayed almost an identical signal; thus, the persulfide sulfur transfer delay was not rescued by Rsm22 overexpression (Figure S4).

An alternative scenario is that Rsm22 alleviates the Yfh1↓ phenotypes not by accelerating persulfide sulfur transfer from Nfs1 to Isu but by enhancing reduction of the persulfide on Isu, i.e., by activating the mitochondrial ferredoxin Yah1. The redox couple ferredoxin (Yah1)/ferredoxin reductase (Arh1) is responsible for reductive conversion of the persulfide to sulfide, thereby converting the sulfur on Isu from 0 to -1 valence, in preparation for combination with Fe^2+^ on Isu to form the nascent [2Fe-2S] cluster [11]. The persulfide reduction by Yah1 represents the first and most immediate step following the frataxin-dependent persulfide sulfur transfer step, and this step could be the site of the Rsm22 bypass activity.

The Yah1 protein was destabilized and turned over in Yfh1↓ mitochondria, perhaps because it is an Fe-S protein, and almost all Fe-S cluster proteins are inactivated in frataxin minus cells [13]. Strikingly, however, the Yah1 protein level was efficiently restored to WT levels by Rsm22 overexpression, even in the absence of Yfh1 (i.e., in Rsm22↑/Yfh1↓ mitochondria) (Figure 8A). In addition, functional data show that forced overexpression of Yah1 in Yfh1↓ mitochondria restored aconitase [4Fe-4S] activity to 90% of the WT level (Figure 8B). Likewise, in the cytoplasm of Rsm22↑/Yfh1↓ cells, the Leu1 isopropylmalate isomerase [4Fe-4S] activity was recovered by more than 90%. Notably, ferredoxin is known to compete with frataxin/Yfh1 for overlapping binding sites on the Nfs1 core complex [7,12,45,46]. Together, we suggest that Rsm22 overexpression might enhance Yah1 stability and/or activity, which, in turn, might allow the Nfs1-bound persulfide sulfur be utilized for Fe-S cluster biosynthesis even in the absence of Yfh1. More work and additional experiments would be needed to establish this hypothetical mechanism for frataxin bypass. The overall process of Fe-S cluster biogenesis is conserved from yeast to humans [5]. In humans, FDX2 is the functional homolog of yeast Yah1 [47]. In the context of frataxin mutations, it is tempting to speculate that altered FDX2 protein stability and/or function could contribute to or modulate different pathologies in clinical settings. Such a finding could then be explored to uncover potentially new therapeutic targets.

## 5. Conclusions

Our findings demonstrate that overexpression of Rsm22 can significantly bypass the mitochondrial dysfunction associated with Yfh1 (frataxin) depletion in yeast (Figure 9). Rsm22 overexpression restores key mitochondrial processes, including Fe-S cluster biosynthesis, lipoic acid synthesis, and iron homeostasis. Importantly, we observed a recovery of cytoplasmic Fe-S cluster assembly, indicating that mitochondrial synthesis and export of (Fe-S)_int_ remain functional. The rescue effect appears to be linked to the stabilization and functional recovery of the mitochondrial ferredoxin Yah1, which is known to compete with Yfh1 for binding to the Nfs1 core complex. These results suggest that Rsm22-mediated frataxin bypass might function through the enhancement of sulfur reduction, a crucial step following persulfide sulfur transfer from Nfs1 to Isu1/2.

While our study provides new insights into alternative pathways for Fe-S cluster assembly in the absence of frataxin, further investigations are necessary to elucidate the precise molecular mechanisms underlying this bypass effect. Future studies should explore whether similar compensatory mechanisms exist in mammalian cells and whether they could be leveraged for therapeutic strategies in Friedreich ataxia.

## Data Availability

The original contributions presented in this study are included in the article/Supplementary Materials. Further inquiries can be directed to the corresponding author.

## Supplementary Materials

The following supporting information can be downloaded at: https://www.mdpi.com/article/10.3390/biom15060785/s1. Figure S1: Rsm22 overexpression restores membrane potential of mitochondria lacking Yfh1; Figure S2: Quantitative analysis of all the Western blots presented in the main manuscript; Figure S3: Comparison of cytoplasmic protein patterns; Figure S4: Evaluating Nfs1-bound persulfide formation in isolated mitochondria; Figure S5: Original Western blot image for Figure 1B; Figure S6: Original Western blot images for Figure 2A and original activity gel for Figure 2B; Figure S7: Original autoradiograph for Figure 3; Figure S8: Original autoradiograph for Figure 4; Figure S9: Original Western blot image for Figure 5B; Figure S10: Original Western blot images for Figure 6A; Figure S11: Original Western blot images for Figure 8A; Figure S12: Original autoradiograph for Figure 8C; Table S1: List of yeast strains used in this study.

## Abbreviations

The following abbreviations are used in this manuscript:

(Fe-S)_int_	(Fe-S) intermediate
ΔNYah1	N-terminal 60 amino acids, including the mitochondrial targeting signal, removed from the Yah1 precursor protein (pYah1)
Leu1^R^	Recombinant Leu1
ISC	Mitochondrial iron–sulfur cluster machinery
CIA	Cytoplasmic iron–sulfur protein assembly machinery
