# Supplementary material for: Salutary Effects of Overexpression of Rsm22, an Assembly Factor for the Mitochondrial Ribosome, on Frataxin/Yfh1 Depletion Phenotypes in Saccharomyces cerevisiae
Source: Biomolecules. 2025 May 28;15(6):785. doi: 10.3390/biom15060785 (PMC12191369; doi:10.3390/biom15060785)
Supplement: Supplementary file 1 [file biomolecules-15-00785-s001.zip › Supplementary Materials/Supplementary Materials.pdf]

Supplementary Materials

Salutary effects of overexpression of Rsm22, an assembly factor for the mitochondrial ribosome, on frataxin/Yfh1 depletion phenotypes in *Saccharomyces cerevisiae*

Ashutosh K. Pandey, Pratibha Singh, Jayashree Pain, Andrew Dancis and Debkumar Pain*

Department of Pharmacology, Physiology and Neuroscience, New Jersey Medical School, Rutgers University, Newark, New Jersey, 07103, USA

Table of contents:

1. Figure S1. Rsm22 overexpression restores membrane potential of mitochondria lacking Yfh1
2. Figure S2. Quantitative analysis of all the Western blots presented in the main manuscript
3. Figure S3. Comparison of cytoplasmic protein patterns
4. Figure S4. Evaluating Nfs1-bound persulfide formation in isolated mitochondria
5. Figure S5. Original Western blot image for Figure 1B
6. Figure S6. Original Western blot images for Figure 2A and original activity gel for Figure 2B
7. Figure S7. Original autoradiograph for Figure 3
8. Figure S8. Original autoradiograph for Figure 4
9. Figure S9. Original Western blot image for Figure 5B
10. Figure S10. Original Western blot images for Figure 6A
11. Figure S11. Original Western blot images for Figure 8A
12. Figure S12. Original autoradiograph for Figure 8C
13. Table S1. List of yeast strains used in this study
14. References

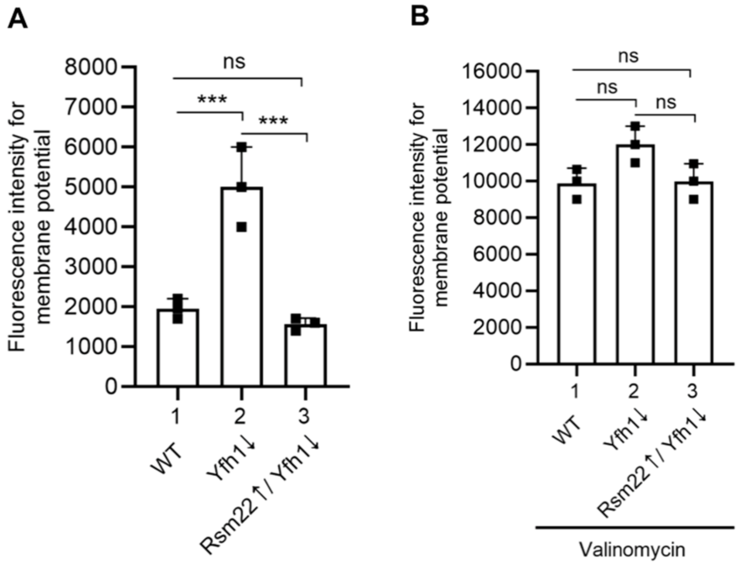

Figure S1. Rsm22 overexpression restores membrane potential of mitochondria lacking Yfh1.

(**A**) Mitochondria were isolated from wild-type (WT), Gal-Yfh1 repressed (Yfh1↓), and Rsm22 overexpressed in Gal-Yfh1 repressed (Rsm22↑/Yfh1↓) cells. Isolated mitochondria (100 μg of proteins) were incubated with a membrane-potential sensitive dye 3,3'-dipropylthiadicarbocyanine iodide (DiSC$_3$(5); 5 μM) in an isotonic buffer (20 mM potassium phosphate pH 7.5, 0.6 M sorbitol, 1% BSA, 10 mM $MgCl_2$, 0.5 mM EDTA). The fluorescence intensity was measured (excitation 622 nm; emission 670 nm) [1]. The reported values are the average of three independent experiments, plotted in GraphPad Prism 10, with error bars representing standard deviation ($P < 0.001$); ns, not significant. (**B**) Same as in (**A**) but mitochondria were pretreated with valinomycin (10 μM). Note that for mitochondria with adequate membrane potential across the inner membrane (negative inside), the cationic dye DiSC$_3$(5) accumulates in the matrix, leading to fluorescence quenching. When the membrane potential is dissipated, the dye is released from mitochondria, exhibiting increased fluorescence intensity.

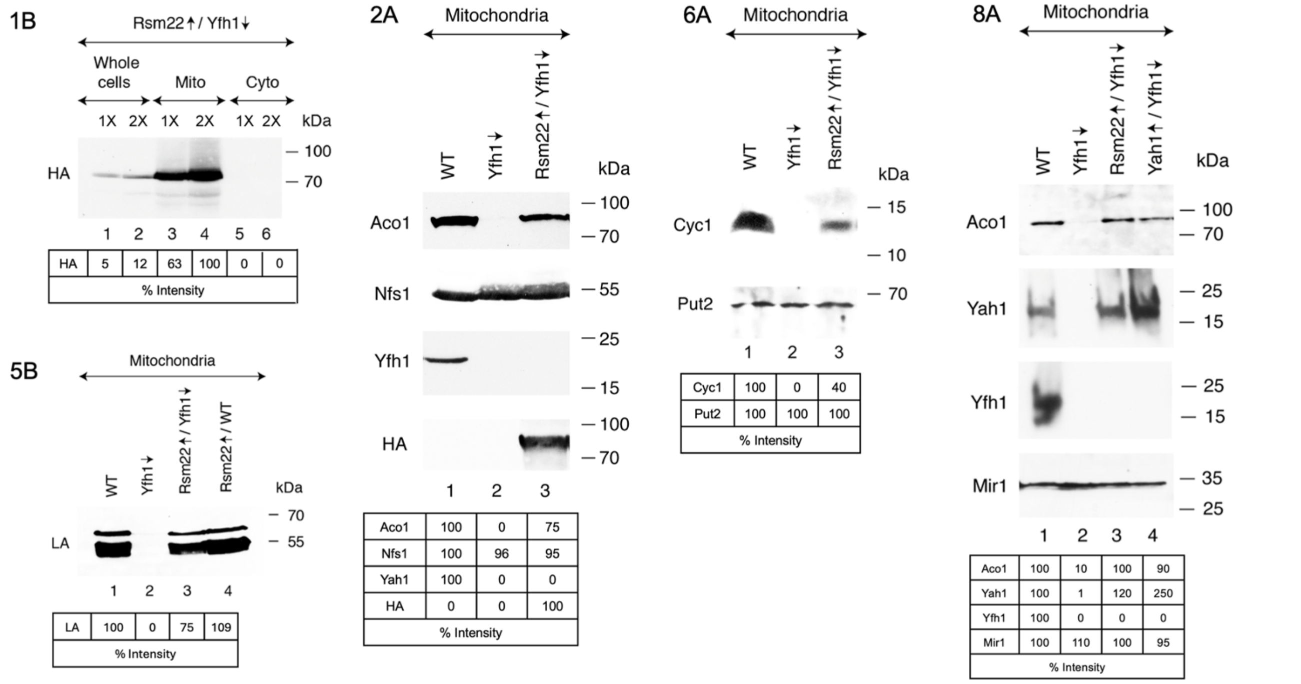

Figure S2. Quantitative analysis of all the Western blots presented in the main manuscript.

Figure 1B) Mitochondrial localization of overexpressed Rsm22-HA3. Mitochondria and cytoplasm were isolated from Rsm22↑/Yfh1↓ cells, and fractions were analyzed by immunoblotting using anti-hemagglutinin (HA) antibodies. Figure 2A) Protein levels. Mitochondrial proteins were analyzed by SDS-PAGE, followed by immunoblotting using antibodies against aconitase (Aco1), cysteine desulfurase (Nfs1), Yfh1, and the HA tag for Rsm22. Figure 5B) Immunoblot. Mitochondrial proteins (200 μg) were analyzed by SDS-PAGE, followed by immunoblotting using antibodies against lipoic acid (LA). Combined anti-LA reactive signals per lane are presented. Figure 6A) Immunoblot. Mitochondrial proteins (200 μg) were analyzed by SDS-PAGE, followed by immunoblotting using antibodies against Cyc1 and Put2. Figure 8A) Protein levels. Mitochondrial proteins were analyzed by SDS-PAGE, followed by immunoblotting using antibodies against Aco1, Yah1, Yfh1, and Mir1.

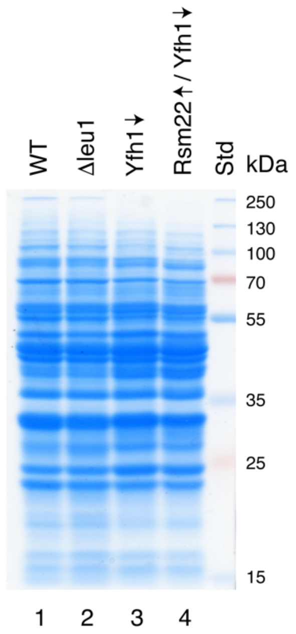

Figure S3. Comparison of cytoplasmic protein patterns. An enriched cytoplasmic fraction was isolated from various yeast strains as indicated. Cytoplasmic proteins (100 µg) were analyzed by SDS-PAGE followed by Coomassie Blue staining. The molecular mass of pre-stained protein standards (Std) is indicated in kDa.

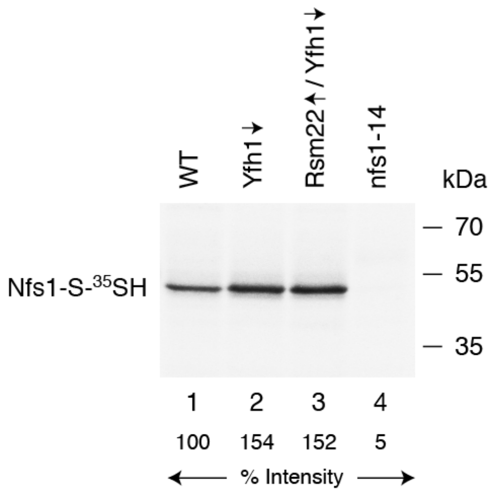

Figure S4. Evaluating Nfs1-bound persulfide formation in isolated mitochondria. As indicated, various mitochondria (100 μg of proteins) were incubated with [^{35}S]cysteine, nucleotides, and iron for 10 min at 25°C, diluted with HS buffer containing 0.15 M NaCl and kept on ice for 10-15 min. After centrifugation, the mitochondrial pellets were analyzed by non-reducing SDS-PAGE, followed by autoradiography. The nfs1-14 mitochondria lack the Nfs1 cysteine desulfurase activity and served as negative control [2].

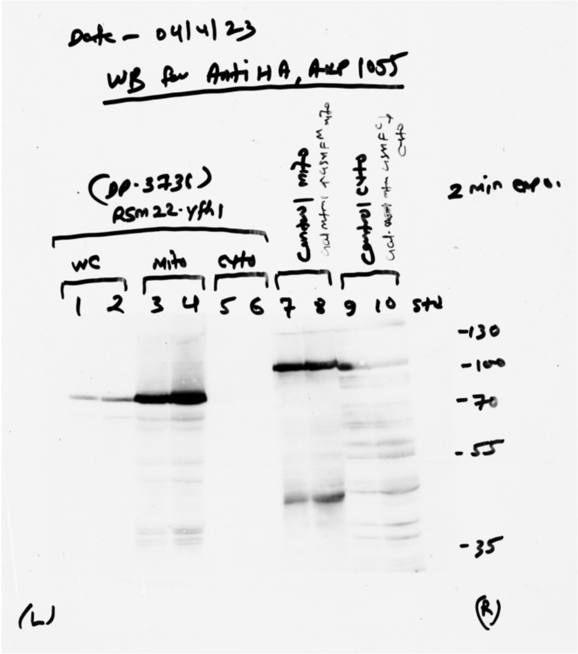

Figure S5. Original Western blot image for Figure 1B (lanes 1-6).

Western blot images

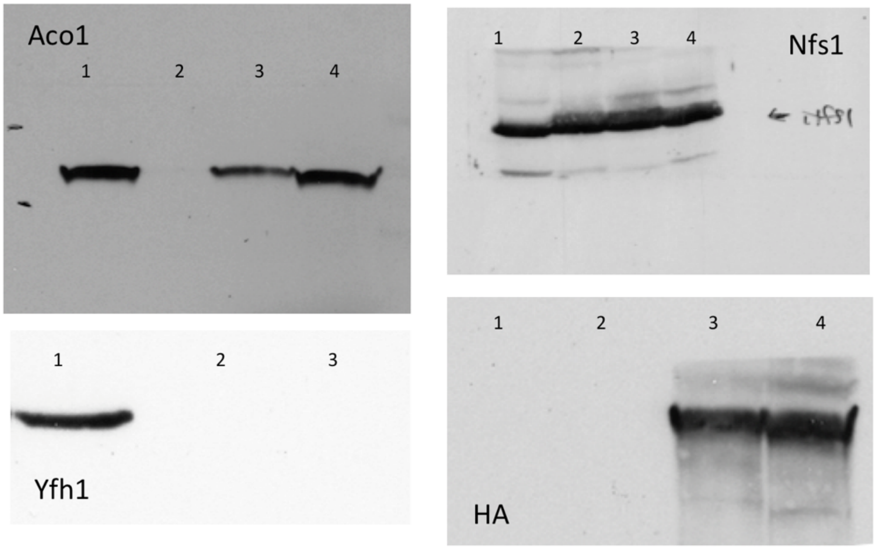

Aconitase in-gel activity

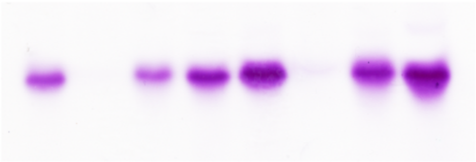

Figure S6. Top panel, original Western blot images for Figure 2A (lanes 1-3). Bottom panel, original activity gel image for Figure 2B (lanes 1-8).

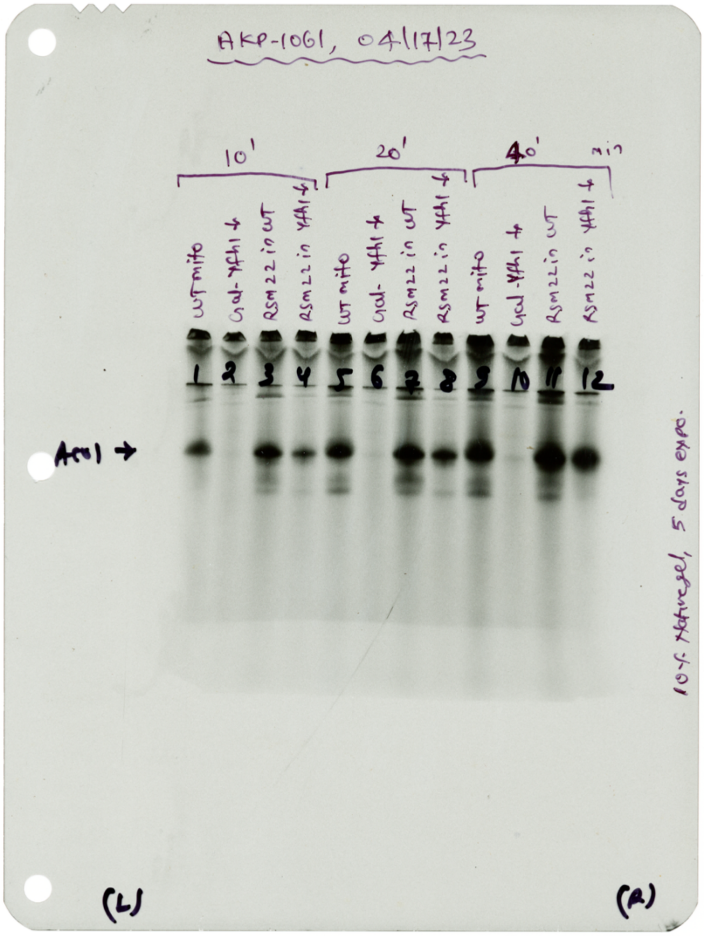

Figure S7. Original autoradiograph for Figure 3 (lanes 1-12).

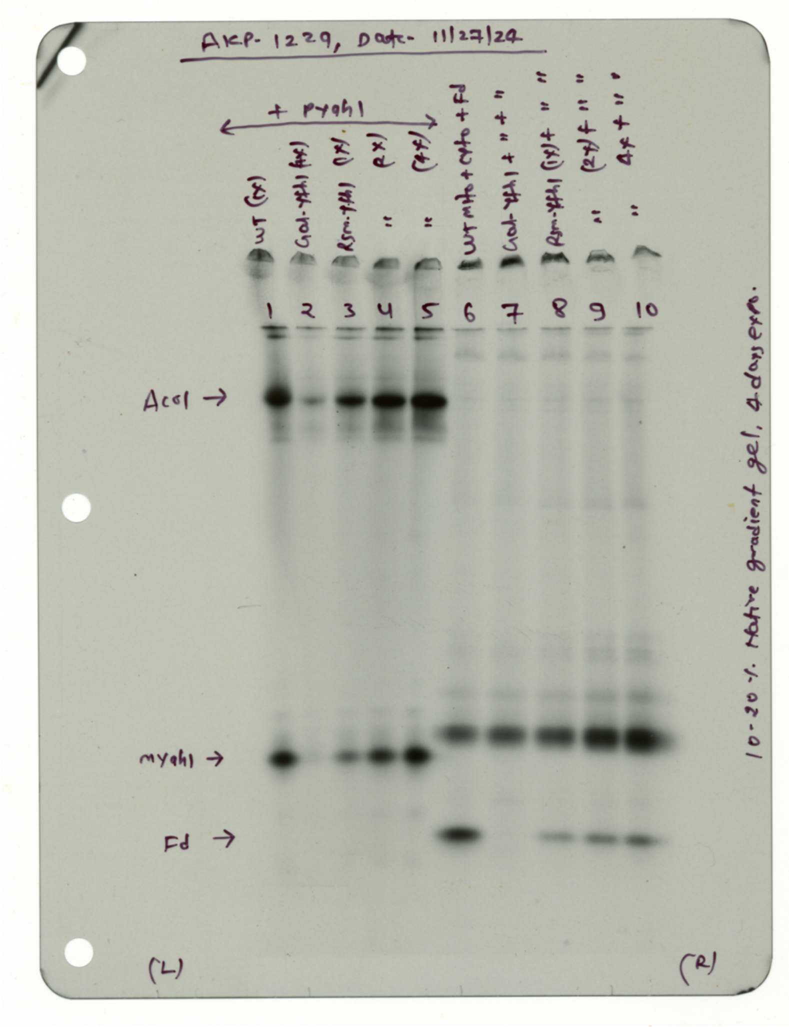

Figure S8. Original autoradiograph for Figure 4 (lanes 1-5).

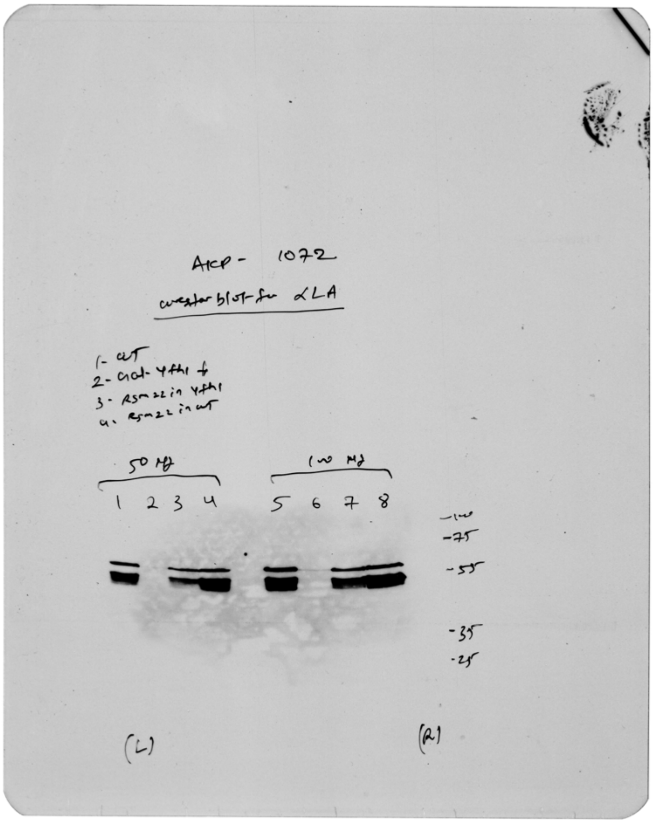

Figure S9. Original Western blot image for Figure 5B (lanes 5-8).

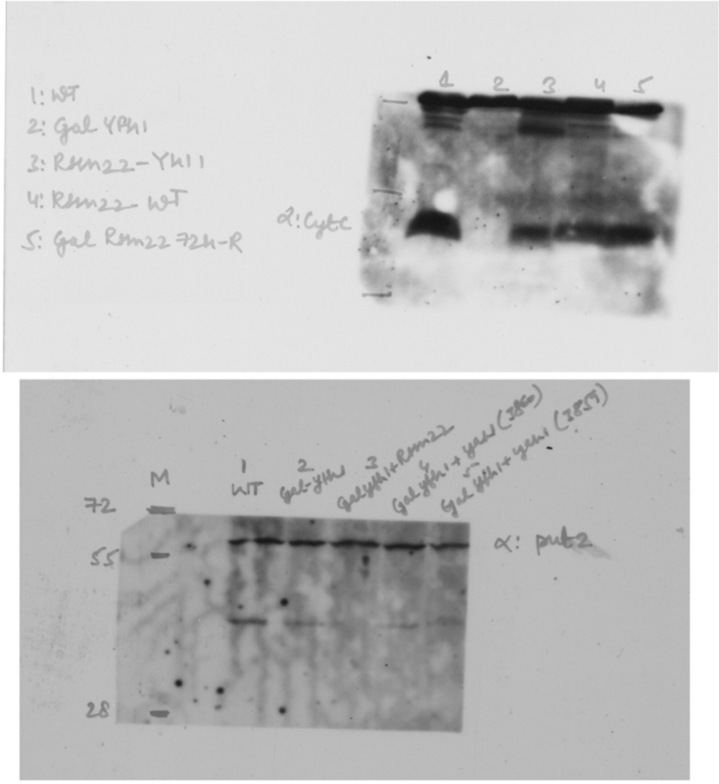

Figure S10. Original Western blot images for Figure 6A (lanes 1-3).

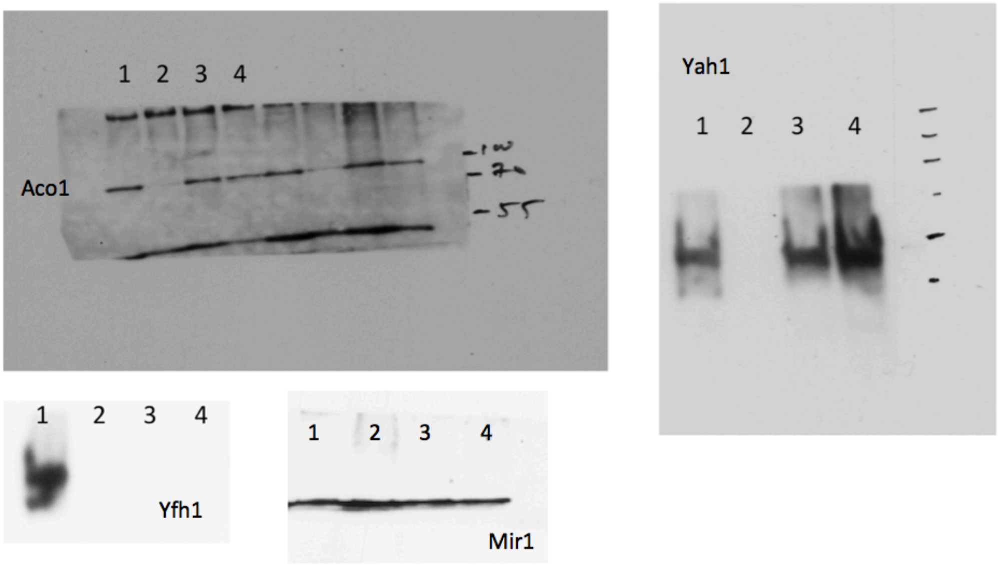

Figure S11. Original Western blot images for Figure 8A (lanes 1-4).

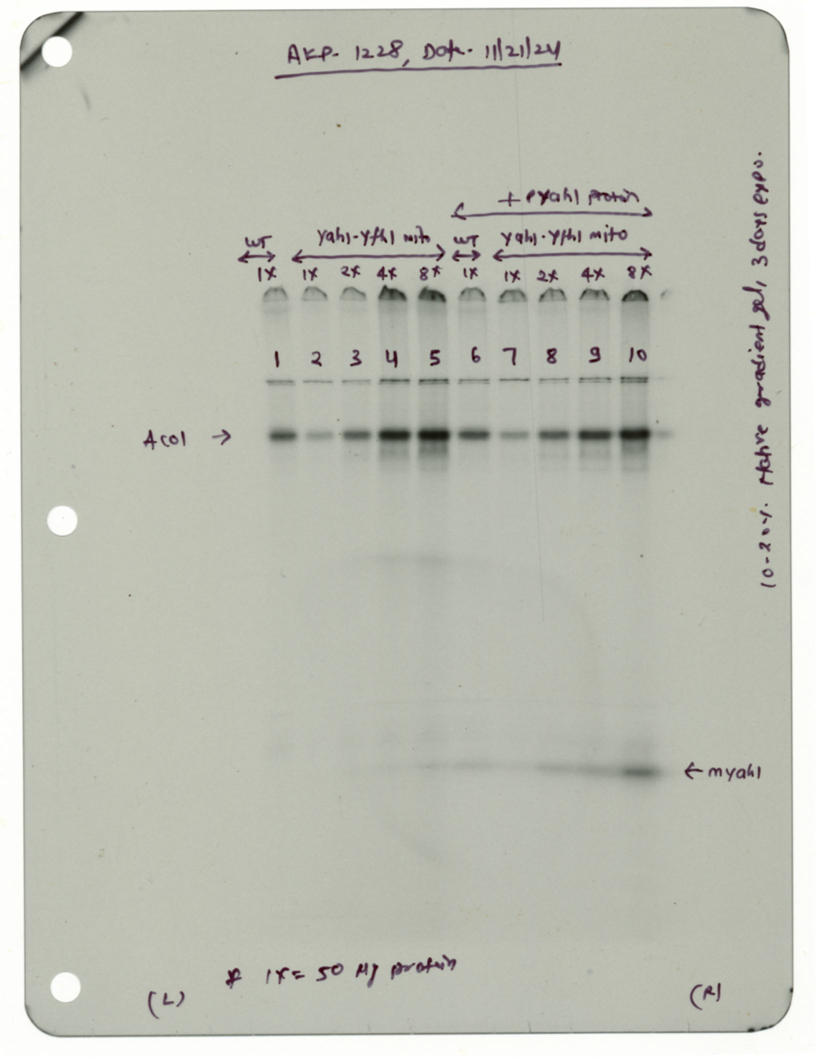

Figure S12. Original autoradiograph for Figure 8C (lanes 1-5).

Table S1. List of yeast strains used in this study

Number	Strain name	Genotype	Source
DP 547	BY4741 (WT)	MATa his3Δ1 leu2Δ0 met15Δ0 ura3Δ0	[3]
DP 3538	BY4741 (Δleu1)	MATa his3Δ1 leu2Δ0 met15Δ0 ura3Δ0 Δleu1::KanMX6	Open Biosystems
AD 99-51	GAL1-Yfh1	MATa his3Δ1 leu2Δ0 met15Δ0 ura3Δ0 His3MX6- PGAL1-YFH1::YFH1	[4]
DP 3736	GAL1-Yfh1 [pRS425-RSM22]	MATa his3Δ1 leu2Δ0 met15Δ0 ura3Δ0 His3MX6- PGAL1-YFH1::YFH1 [pRS425-GPDprom-RSM22-HA3]	This work
DP 3740	WT [pRS425-RSM22]	MATa his3Δ1 leu2Δ0 met15Δ0 ura3Δ0 [pRS425- GPDprom-RSM22-HA3]	This work
DP 3860	GAL1-Yfh1 [pRS425-YAH1]	MATa his3Δ1 leu2Δ0 met15Δ0 ura3Δ0 His3MX6- PGAL1-YFH1::YFH1 [pRS425-GPDprom-YAH1-His6]	This work

References

1. Geissler, A.; Krimmer, T.; Bomer, U.; Guiard, B.; Rassow, J.; Pfanner, N. Membrane potential-driven protein import into mitochondria. The sorting sequence of cytochrome b_2 modulates the delta psi-dependence of translocation of the matrix-targeting sequence. *Mol. Biol. Cell* **2000**, *11*, 3977-3991.
2. Li, J.; Kogan, M.; Knight, S.A.; Pain, D.; Dancis, A. Yeast mitochondrial protein, Nfs1p, coordinately regulates iron-sulfur cluster proteins, cellular iron uptake, and iron distribution. *J. Biol. Chem.* **1999**, *274*, 33025-33034.
3. Pandey, A.K.; Pain, J.; Singh, P.; Dancis, A.; Pain, D. Mitochondrial glutaredoxin Grx5 functions as a central hub for cellular iron-sulfur cluster assembly. *J. Biol. Chem.* **2025**, *301*, 108391.
4. Yoon, H.; Golla, R.; Lesuisse, E.; Pain, J.; Donald, J.E.; Lyver, E.R.; Pain, D.; Dancis, A. Mutation in the Fe-S scaffold protein Isu bypasses frataxin deletion. *Biochem. J.* **2012**, *441*, 473-480.
